# Genetic proxies for antihypertensive drugs and mental disorders: Mendelian randomization study in European and East Asian populations

**DOI:** 10.1186/s12916-023-03218-6

**Published:** 2024-01-02

**Authors:** Bohan Fan, Jie V. Zhao

**Affiliations:** School of Public Health, Li Ka Shing Faculty of Medicine, The University of Hong Kong, 1/F, Patrick Manson Building, 7 Sassoon Road, Hong Kong SAR, China

**Keywords:** Antihypertensive drugs, Mental disorders, Mendelian randomization

## Abstract

**Background:**

Mental disorders are among the top causes of disease burden worldwide. Existing evidence regarding the repurposing of antihypertensives for mental disorders treatment is conflicting and cannot establish causation.

**Methods:**

We used Mendelian randomization to assess the effects of angiotensin-converting-enzyme inhibitors (ACEIs), beta blockers (BBs), and calcium channel blockers (CCBs) on risk of bipolar disorder (BD), major depression disorder (MDD), and schizophrenia (SCZ). We used published genetic variants which are in antihypertensive drugs target genes and correspond to systolic blood pressure (SBP) in Europeans and East Asians, and applied them to summary statistics of BD (cases = 41,917; controls = 371,549 in Europeans), MDD (cases = 170,756; controls = 329,443 in Europeans and cases = 15,771; controls = 178,777 in East Asians), and SCZ (cases = 53,386; controls = 77,258 in Europeans and cases = 22,778; controls = 35,362 in East Asians) from the Psychiatric Genomics Consortium. We used inverse variance weighting with MR-Egger, weighted median, weighted mode, and Mendelian Randomization Pleiotropy RESidual Sum and Outlier. We performed gene-specific analysis and utilized various methods to address potential pleiotropy.

**Results:**

After multiple testing correction, genetically proxied ACEIs were associated with an increased risk of SCZ in Europeans (odds ratio (OR) per 5 mmHg lower in SBP 2.10, 95% CI 1.54 to 2.87) and East Asians (OR per 5 mmHg lower in SBP 2.51, 95% CI 1.38 to 4.58). Genetically proxied BBs were not associated with any mental disorders in both populations. Genetically proxied CCBs showed no benefits on mental disorders.

**Conclusions:**

Antihypertensive drugs have no protection for mental disorders but potential harm. Their long-term use among hypertensive patients with, or with high susceptibility to, psychiatric illness needs careful evaluation.

**Supplementary Information:**

The online version contains supplementary material available at 10.1186/s12916-023-03218-6.

## Background

Mental disorders including bipolar disorder (BD), major depression disorder (MDD), and schizophrenia (SCZ) remained among the leading causes of disease burden worldwide over the past two decades [1]. Existing antipsychotic medications are effective in treating symptoms, but undesired side effects including akathisia and weight gain may lead to poor adherence [2], and their long-term use may adversely affect cardiovascular disease risk and risk factors [3]. Hypertension is prevalent among patients with bipolar disorder and anxiety [4] and hypertensive patients are more likely to experience negative emotions such as anxiety, stress, and depression [5]. Antihypertensive drugs such as angiotensin-converting enzyme inhibitors (ACEIs), beta-blockers (BBs), and calcium channel blockers (CCBs) have been investigated for their repurposing opportunities in mental disorders [6]. For example, ACEIs that act on the renin-angiotensin system (RAS) may exert protective effects on cognition, depression, and anxiety [7]. BBs may have antidepressant and anti-anxiety effects [7]. CCBs which target L-type voltage-gated calcium channels are known to play an important role in fundamental neuronal processes related to psychiatric disorders [8]. Genomic studies also showed the single-nucleotide polymorphisms (SNPs) in the cross-disorder risk gene, *CACNA1C*, was related to the risk of psychiatric prognosis [9, 10].

Currently, epidemiological evidence remains inconclusive with conflicting findings reported. High-quality randomized controlled trials (RCTs) are limited. Observational studies have found that antihypertensive drug classes were differentially related to the incidence of psychiatric disorders [11]. Recent Mendelian randomization (MR) assessing the association between gene expression of 22 antihypertensive drug target genes has shown an adverse association of *ACE* gene with SCZ risk in the European population, but did not focus on the effect of antihypertensive drugs, especially the effect of antihypertensive drugs with multiple target genes [12]. In addition, the associations of different antihypertensive drugs in other populations, such as East Asians, have not been examined. A previous systematic review of double-blind RCTs suggested BBs do not affect the risk of depression [13]. Two European observational studies reported CCBs could reduce psychiatric hospitalization and self-harm in individuals with BD [14, 15], whereas systematic reviews and meta-analyses based on a few small and low-quality RCTs suggested CCBs were not effective in treating BD [16, 17]. A Scottish observational study using linkage healthcare data did not find any antihypertensive drugs to prevent the new onset of MDD [18]. So far, there is limited evidence on the use of antihypertensives for BD, MDD, and SCZ, and whether the observed associations were causal is unclear.

Under this circumstance, MR offers unique opportunities to strengthen causal inference. MR utilizes genetic variants inherited at birth as an instrument, thus it is not prone to confounding [19]. It has been applied to examine the effects of antihypertensive drugs on various outcomes [20], such as diabetes [21], stroke [22], and kidney functions [23]. Using MR for investigating drug effects not only facilitates pharmaceutical companies with a more cost-effective drug development process but also helps clinicians make better prescription decisions for comorbid individuals. Here, we explored the effects of antihypertensive drugs on BD, MDD, and SCZ in both European and East Asian populations.

## Methods

### Study design

We assessed the effects of major antihypertensive drugs ACEIs, BBs, and CCBs on BD, MDD, and SCZ using two-sample MR. We obtained genetic variants of antihypertensive drug target genes as instruments from previously published studies [20–24]. We obtained genetic associations with systolic blood pressure (SBP) from meta-analysis of large genome-wide association studies (GWAS) in the European population, and from the Biobank Japan in the East Asian population, and applied them to mental disorders GWAS from Psychiatric Genomics Consortium (PGC). MR requires the fulfilment of three assumptions. First, the genetic instruments must be associated with exposure (the relevance assumption). Second, the genetic instruments must not be associated with confounders (the independent assumption). Third, the genetic instruments must be independent of the outcome given the exposure (the exclusion-restriction assumption) [19]. A flow chart of the overall study design is presented in the Additional file 1: Supplementary Methods.

### Genetic instruments of exposure

We used genetic variants that mimic the effect of lowering SBP through antihypertensive drug targets chosen in corresponding genes for ACEIs, BBs, and CCBs from published studies [20, 21, 23]. Specifically, *ACE* gene for ACE inhibitors*, ADRB1* gene for BBs, and 11 genes for CCBs (*CACNA1D, CACNA1F, CACNA2D1, CACNA2D2, CACNA1S, CACNB1, CACNB2, CACNB3, CACNB4, CACNG1,* and *CACNA1C*) that encode the targets of these drugs related to effects on blood pressure that were identified using the DrugBank database [20]. SNPs that are located near (± 200 kb) or within these drug target genes, and associated with SBP at genome-wide significance (*p* < 5 × 10^–8^) in a large GWAS of SBP were selected, and clumped using a linkage disequilibrium (LD) threshold of *r*^2^ < 0.1 with reference to the 1000G European reference panel for Europeans and the East Asian 1000 Genomes panel for East Asians (Additional file 1: Table S1). For the European population, genetic associations with SBP were obtained from a meta-analysis of the UK Biobank and the International Consortium of Blood Pressure GWAS (*N* = 757,601), adjusted for age, age^2^, sex, body mass index (BMI), principal components and study-specific covariates [25]. We also obtained the association of the genetic proxies for antihypertensive drugs with SBP using the GWAS of SBP from the UK Biobank, with no adjustment for BMI (GWAS id: ukb-b-20175). For the East Asian population, we used genetic variants from a previous study [21], that are located near (± 200 kb) or within these drug target genes, and also associated with SBP in a GWAS in the Biobank Japan (*n* = 136,597) [26]. The GWAS adjusted for age, sex, top ten principal components of genetic ancestry, disease status, and any necessary trait-specific covariates. In sensitivity analysis, as previously [23], we used genetic variants regulating the expression of the relevant drug target genes identified from expression quantitative trait loci (eQTL), and selected genetic variants related to SBP in the UK Biobank [23, 27]. Only SNPs with F-statistics above 10 were used [23]. All effect estimates were scaled to effect corresponding to a 5-mmHg lower in SBP. We looked up all SNPs in Phenoscanner (http://www.phenoscanner.medschl.cam.ac.uk/), a curated database platform with genotype–phenotype associations to check for associations with potential confounders including BMI, education, Townsend deprivation index, tobacco smoking, and alcohol intake. We also checked for their association with survival, which may lead to survival bias.

### Outcomes

We obtained genetic associations with mental disorders from PGC. BD was conducted with 41,917 cases (BD type I and II) and 371,549 controls in the European population only [28]. Genetic associations with MDD were obtained from summary statistics in a meta-analysis of the 33 cohorts [29] from the European countries (excluding UK Biobank and 23andMe data)[30], with a total number of 500,199 individuals (170,756 cases and 329,443 controls). SCZ was conducted within 90 cohorts, of which 80% were of European ancestry (53,386 cases and 77,258 controls) [31]. In East Asians, we did not identify GWAS of BD. Genetic associations with MDD were obtained from a meta-analysis of the nine largest published GWAS for MDD among individuals of East Asian descent (mean age ~ 51.3 years; 62.8% women), which included 15,771 individuals with depression and 178,777 controls [32]. SCZ in East Asians was conducted among 22,778 cases and 35,362 controls [33].

### Statistical analysis

We obtained the Wald-ratio estimate for each SNP (i.e., the genetic association with the risk of mental disorder divided by the genetic association with the genetic proxies for exposure) and meta-analyzed SNP-specific estimates using multiplicative random-effects inverse variance weighting (IVW) in main analysis. In harmonization, we ensured the association of each SNP with the exposure and with the outcome corresponds to the same allele. We also checked effect allele frequency for palindromic SNPs, i.e., the alleles are C/G (or A/T) with 0.42 < effect allele frequency < 0.58, and dropped SNPs with ambiguous strand reported [34]. To address potential pleiotropy, we used different methods including the MR-Egger regression, weighted median, weighted mode, and the Mendelian randomization pleiotropy residual sum and outlier (MR-PRESSO). MR-Egger provides unbiased estimates to allow for directional pleiotropy but still requires the pleiotropic effects to be independent of the instrument strength (the ‘InSIDE’ assumption) [35]. When a non-zero MR-Egger intercept indicates potential horizontal pleiotropy exists, we used MR-Egger estimates instead of IVW estimates and assessed the ‘NO Measurement Error’ (NOME) assumption with I^2^_GX_ indicator to test NOME violation [36]. The weighted median requires at least 50% of the weight comes from valid instruments to give a consistent estimation [37]. The weighted mode estimation assumes a plurality of genetic variants to be valid [38]. Also, the presence of pleiotropic outliers was tested with MR-PRESSO, which provides corrected estimates after removing outlier SNPs [39]. To account for multiple testing, the association with a Bonferroni corrected *p*-value < 0.05/15 = 0.0033 (15 = 3 drugs × 3 outcomes in Europeans + 3 drugs × 2 outcomes in East Asians) was considered as statistically significant. Associations with *p*-value < 0.05 but did not reach Bonferroni-corrected significance (i.e., nominal significance) were considered as suggestive evidence.

In sensitivity analyses, we used genetic instruments (8 SNPs for CCBs and 2 SNPs for BBs in the European population as shown in Additional file 1: Table S1) selected from a more stringent LD threshold of *r*^2^ < 0.01, as previously [20, 23]. We conducted a gene-specific analysis for CCBs with mental disorders to entangle the effects of individual genes using random-effects IVW to aggregate genetic variants related to each gene as instruments. The number of genetic instruments used for each gene is presented in the Additional file 1: Table S1. Further to address potential horizontal pleiotropy in CCBs, we did the following sensitivity analysis: 1) we removed SNPs correspond to *CACNA1C* gene for Europeans [9, 10] and *CACNB2* gene for East Asians [40] that may link to mental disorders; 2) we removed SNPs related to BMI, a potential confounder or a mediator [41]; 3) we removed SNPs related to cause of death considering the potential survival bias; 4) we removed all pleiotropic SNPs for CCBs; 5) we removed pleiotropic SNPs from SNPs selected with LD of *r*^2^ < 0.01. Given that MR could be biased due to confounding by LD when two distinct genetic variants are correlated with each other, which violates the exclusion-restriction assumption, we performed Bayesian colocalization for ACEIs with mental disorders when the associations passed Bonferroni-corrected significance. Colocalization compares the genetic associations for two traits at the same gene region [42]. In colocalization, we assessed the following hypotheses: H_0_: no association with either trait; H_1_: association with only trait 1; H_2_: association with only trait 2; H_3_: two independent SNPs associated with both traits; H_4_: the same SNP associated with both traits [42]. We consider the posterior probability for shared causal variants (PP_H4_) > 0.8 to have strong evidence of colocalization while a high PP_H3_ suggests confounding by LD [42]. To further enhance the robustness of the findings in consideration of diastolic blood pressure (DBP), we repeated the analyses with DBP-associated SNPs using a similar strategy to select instrument for SBP-associated SNPs in main analysis. The information of genetic instruments of antihypertensive drugs and their associations with DBP are provided in the Additional file 1: Table S1. Power calculation was based on the approximation that the sample size of an MR study is the sample size for exposure on outcome divided by the R^2^ for genetic instruments on exposure (Additional file 1: Table S2) [43]. All statistical analyses were performed using R (v4.0.1, the R Foundation for Statistical Computing, Austria) and the packages ‘Mendelian randomization (v0.7.0)’, ‘TwoSampleMR (v0.5.6)’, ‘coloc (v5.2.2)’ and ‘MR-PRESSO (v1.0)’.

## Results

**Fig. 1:**
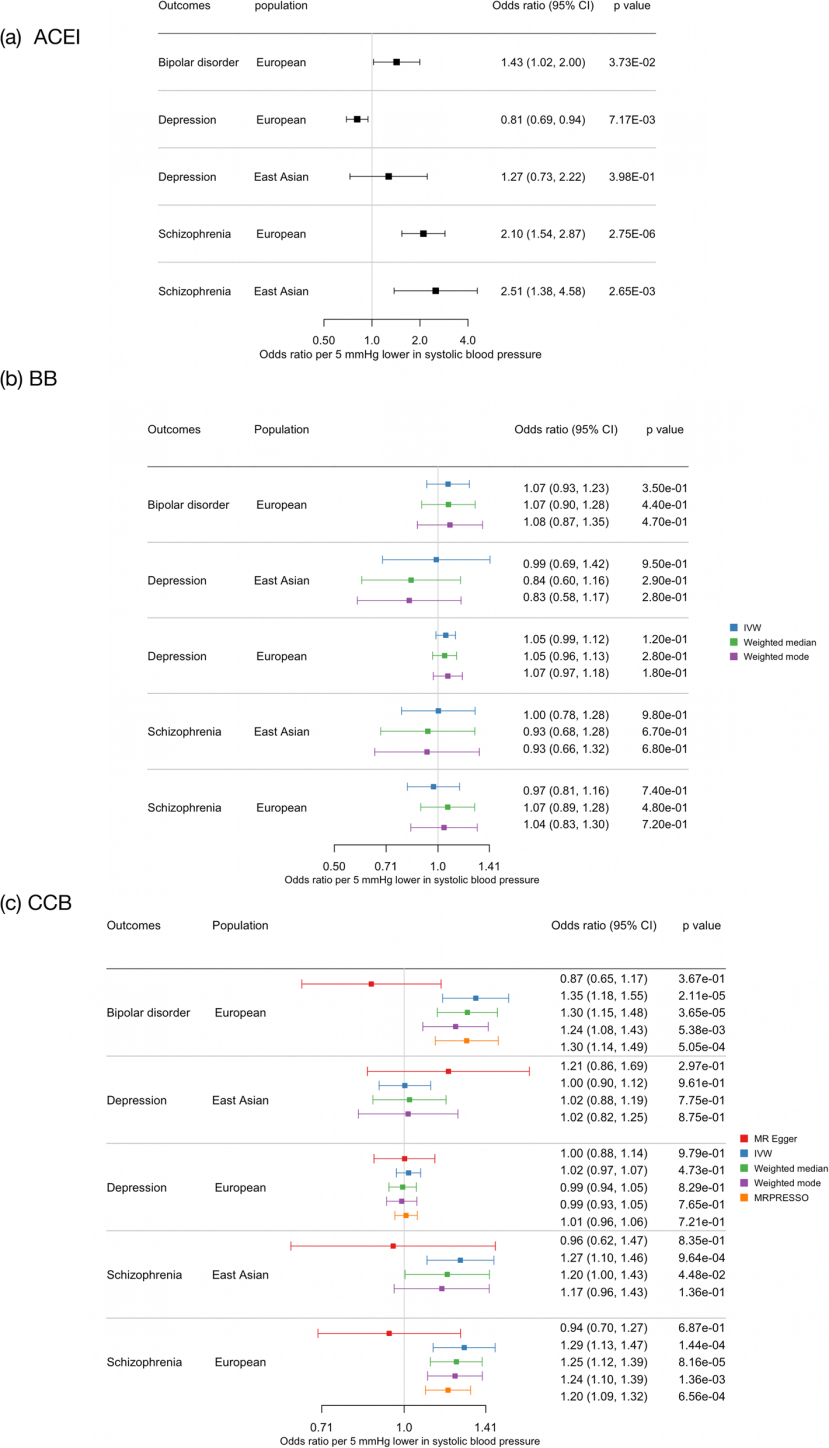
Associations of genetically proxied angiotensin-converting-enzyme inhibitors (ACEIs), beta blockers (BBs), and calcium channel blockers (CCBs) with mental disorders

Genetically proxied ACEIs were associated with an increased risk of SCZ in Europeans (OR per 5 mmHg lower in SBP 2.10, 95% CI 1.54 to 2.87) and East Asians (OR per 5 mmHg lower in SBP 2.51, 95% CI 1.38 to 4.58) (Fig. 1a). Genetically proxied ACEIs were nominally associated with BD (OR per 5 mmHg lower in SBP 1.43, 95% CI 1.02 to 2.00) and MDD in Europeans (OR per 5 mmHg lower in SBP 0.81, 95% CI 0.69 to 0.94). Using IVW, weighted median, and weighted mode, we consistently observed null associations between BBs and mental disorders in both populations (Fig. 1b) and results were consistent using a more stringent instrument selection LD threshold (Additional file 1: Fig. S1) or using the set of eQTL SNPs as genetic instruments (Additional file 1: Table S3). For CCBs, three SNPs (rs3821843, rs10828399, and rs61842677) were related to BMI and two SNPs (rs113210396 and rs72786098) were related to cause of death. We did not find a beneficial role of genetically proxied CCBs on mental disorders and we cannot exclude an association of CCBs with the risk of BD and SCZ (Fig. 1c, Additional file 1: Fig. S1-S6). The MR-Egger intercept does not equal to zero suggested possible horizontal pleiotropy in the effect of CCBs on BD and SCZ (Additional file 1: Fig. S8), and corrected estimates from MR-Egger showed null association (Fig. 1c). The I^2^_GX_ values were above 0.9, indicating that the "NOME" assumption has been fulfilled for all MR-Egger analyses (Additional file 1: Table S4). We found consistent, positive associations of CCBs with BD and SCZ while null association with MDD using weighted median and weighted mode methods, after removing SNPs related to *CACNA1C* gene in the Europeans and *CACNAB2* gene in the East Asian population (Additional file 1: Fig. S2), after removing SNPs related to BMI (Additional file 1: Fig. S3), and after removing SNPs related to cause of death for the European population (Additional file 1: Fig. S4). The positive associations with BD (OR per 5 mmHg reduction in SBP 1.32, 95% CI 1.15 to 1.52) and SCZ (OR per 5 mmHg reduction in SBP 1.21, 95% CI 1.09 to 1.34) in the Europeans remained and there was no indication of horizontal pleiotropy after excluding all these pleiotropic SNPs (Additional file 1: Fig. S5). Similar results were found using genetic instruments selected from a more stringent LD threshold when excluding all pleiotropic SNPs (Additional file 1: Fig. S6).

In the gene-specific analysis for CCBs, *CACNA1C* (based on two SNPs) was associated with a higher risk of BD (OR per 5 mmHg reduction in SBP 2.54, 95% CI 1.75 to 3.68) and SCZ (OR per 5 mmHg reduction in SBP 2.88, 95% CI 2.01 to 4.13) in the European population, while *CACNAB2* (based on seven SNPs) was associated with a higher risk of SCZ (OR per 5 mmHg reduction in SBP 1.49, 95% CI 1.03 to 2.13) and a lower risk of MDD (OR per 5 mmHg reduction in SBP 0.76, 95% CI 0.6 to 0.97) in East Asians (Additional file 1: Fig. S7). Nevertheless, MR analysis using genetic instruments excluding the SNPs on *CACNA1C* in Europeans and related to *CACNAB2* in East Asians still showed similar associations, suggesting that the observed drug effect may not be fully driven by these genes (Additional file 1: Fig. S2). In addition, the analysis using the GWAS of SBP from the UK Biobank with no adjustment for BMI provides similar estimates as the main results (the Additional file 1: Table S5). The additional analysis using DBP-associated SNPs also supports the robustness of our main findings (Additional file 1: Table S7). In colocalization, there is evidence of colocalization with SCZ in Europeans (PP_H4_ = 0.98), the low posterior probability for the distinct causal variants (PP_H3_ = 0.01) suggests a low probability of confounding by LD. Similarly, we found a low probability of confounding by LD for SCZ in Asians with PP_H3_ = 0.05 and PP_H4_ = 0.35 (Additional file 1: Fig. S9).

## Discussion

Our study showed that genetically proxied ACEIs were associated with an elevated risk of SCZ. Genetically proxied BBs were not associated with any mental disorders in both populations. Using various sensitivity analyses to address potential pleiotropy, we examined the effect of genetically mimicked CCBs, reflected by SBP reduction, on mental disorders. Our study does not support a beneficial role of CCBs on mental disorders but raises the possibility that CCBs might increase the risk of BD and SCZ. Gene-specific analysis for CCBs suggested that *CACNA1C* in the European population could raise BD and SCZ risk, while *CACNAB2* in East Asians may increase SCZ risk but lower MDD risk. Mental health is under-recognized in hypertension clinical practice. This study adds to the limited evidence regarding the possible impact that antihypertensive drugs have on mental disorders in both European and East Asian populations, by showing that genetically proxied ACEIs were associated with an increased risk of SCZ in Europeans and East Asians, and genetically proxied CCBs showed no benefits but potential harm on BD and SCZ in Europeans.

The observed adverse effect of ACEIs on SCZ in the European population is consistent with the previous MR study showing *ACE* gene expression was associated with SCZ risk [12]. Our findings using the set of eQTL SNPs as genetic instruments and their genetic associations with SBP provides evidence from another perspective. As the effect of antihypertensive drugs is reflected by the extent of lowering blood pressure, using the genetic associations with SBP can also provide a more straightforward interpretation. Regarding the association of ACEIs with MDD, there is suggestive evidence that genetically proxied ACEIs were associated with lower MDD risk in the Europeans while the association in the East Asians was null. However, the difference between populations was not statistically significant (*p* value for the difference = 0.16). The discrepancy between populations could be due to lack of power in the East Asians. Hence, the results need replication if larger GWAS in East Asians is available in the future. ACEIs may not be suitable for repositioning for mental disorders. Null effect was found between BBs and mental disorders in both populations, consistent with previous systematic reviews of RCTs [13]. Our result on CCBs is consistent with an observational study that showed null associations with SCZ, BD and MDD [6], but contradicts with others [11, 14, 18]. The discrepancy could be due to potential confounding by environmental and socioeconomic factors in observational studies. Interestingly, the gene-specific effects of CCBs differed by population. For example, *CACNA1C* is harmful in Europeans but not in East Asians, while *CACNB2* is associated with a higher risk of mental disorders in East Asians [40]. While we found *CACNB2* is associated with increased risk of BD and SCZ, the previous study found null association of *CACNB2* gene expression with these diseases [12]. The discrepancy is possibly because the previous study used the association with gene expression as exposure, while we used the association with SBP reduction as exposure. BBs and CCBs may not be considered as new drug candidates for psychiatric use as well.

Potential mechanisms could explain the observed associations of antihypertensive drugs with mental disorders. An animal study demonstrated that the brain RAS of which ACEIs target on, could modulate multiple brain functions such as sensory information processing, learning, memory, and emotional responses [7]. The possible explanation for the association of ACEIs with SCZ is that ACE and the central RAS could play a role in inflammation and immunity [44], and immune dysfunction might contribute to the pathogenesis of schizophrenia [45]. The link between inflammation and psychiatric disorders has also been found in CCBs, which might increase TNF-a, an inflammatory factor [46] and inflammation may be associated with schizophrenia [47]. A possible mechanism linking CCBs and SCZ could relate to calcium influx, which is crucial for multiple neuronal processes [8].

Although MR can minimize residual confounding and utilize sufficient power, our study has several limitations. First, as weak instruments may bias towards the null, we selected strong genetic variants as instrumental variables with an F-statistic > 10. Horizontal pleiotropy may bias MR estimates, however, we addressed this by conducting various analytic methods and sensitivity analyses. To mitigate the bias due to confounding by LD, we showed similar results using different LD clumping threshold strategies in main analysis and sensitivity analysis. Colocalization also suggested that the association is less likely due to confounding by LD and supported the validity of the results. Second, we used MR to explore overall lifelong effects of antihypertensive medication use on mental disorders instead of their short-term use, hence, results from short-term observational studies were not comparable. The MR estimates may have overestimated the drug effects because MR examines the lifelong effects while the use of drugs is within a limited period. However, the directions of associations can provide suggestions on the possible off-target effects of antihypertensive drugs. Third, genetic variations only explain a small proportion of the exposure, however, we leveraged large GWAS summary data to maximize power. Fourth, additional MR assumptions require linearity of exposure, but the assumption is not testable using summary data. Fifth, developmental compensatory processes such as canalization can occur, so the expected effect of genetic change might be reduced. Sixth, GWAS of BD have partially overlapping samples with the exposure GWAS (e.g., UK Biobank) that might inflate type I error rate in two-sample MR [48], sensitivity analysis using genetic associations with BD derived from non-overlapping data of the same study provided similar estimates (Additional file 1: Table S6). Seventh, we found different antihypertensive drugs may play different roles on mental disorders, which suggests that the effect may not be fully via lowering SBP, other pathways may exist. As we used SBP reduction to reflect the effect of antihypertensive drugs, we cannot perform multivariable MR to test the effect independent of SBP. Lastly, genetic instruments are proxies for antihypertensive drug classes, they cannot distinguish between the antihypertensives subclasses (i.e., dihydropyridine v.s. non-dihydropyridine CCBs). In addition, the genetic interrogation of drug target effects cannot be simply extended to clinical practice without consideration of the pharmacokinetics of drugs, such as blood–brain barrier penetrability.

## Conclusions

In summary, antihypertensive drugs showed differential effects on the risk of mental disorders. Our study does not support a protective effect of genetically proxied ACEIs, BBs, or CCBs on mental disorder risks in both European and East Asian populations, so they may not be suitable for repurposing. Future studies need to clarify whether antihypertensive drugs like ACEIs and CCBs would exert adverse psychiatric effects. From a clinical perspective, it is important to understand whether antihypertensive drugs may cause, exacerbate, or relieve psychiatric symptoms so that clinicians can provide better treatment in comorbid individuals, especially hypertensive patients with, or with high susceptibility to, psychiatric illness.

### Supplementary Information

**Additional file 1:** **Supplementary Methods.** Flow chart of instrument selection and study design. **Figure S1.** Associations of BBs and CCBs using SNPs selected from LD threshold at r^2^< 0.01 in the European population. Two SNPs were used for genetic proxies for BBs and 8 SNPs were used for genetic proxies for CCBs. **Figure S2.** MR analysis for CCBs excluding pleiotropic SNPs related to *CACNA1C *gene in the European population and excluding pleiotropic SNPs related to *CACNAB2* gene in the East Asian population. 22 SNPs served as genetic instruments in the European population and 14 SNPs served as genetic instruments in the East Asian population. **Figure S3.** MR analysis for CCBs excluding pleiotropic SNPs related to BMI. After removing rs3821843 and rs10828399 for Europeans and rs61842677 for East Asians related to BMI, 22SNPs served as genetic instruments in the European population and 20 SNPs served as genetic instruments in the East Asian population. **Figure S4.** MR sensitivity analysis for CCBs excluding pleiotropic SNPs related to cause of death. After removing two SNPs (rs113210396 and rs72786098) related to cause of death for the European population, 22SNPs served as genetic instruments for CCBs. **Figure S5.** MR sensitivity analysis for CCBs excluding pleiotropic SNPs related to BMI, *CACNA1C* gene, and cause of death. After removing all six SNPs related to *CACNA1C*, BMI and cause of death for the European population, 18 SNPs served as genetic instruments for CCBs. **Figure S6.** Associations of CCBs using SNPs selected from LD threshold r^2^< 0.01 and excluded pleiotropic SNPs related to BMI and *CACNA1C* gene. After removing 2 SNPs related to risk genes and potential confounder BMI for the European population, 6 SNPs served as genetic instruments. **Figure S7.** MR analysis of gene-specific effects of CCBs. Nsnp (column 4) indicates the number of SNPs aggregated for each gene targeted region of CCBs. **Figure S8.** Visual representation of the MR-Egger estimates of the genetic associations of CCBs with BD and SCZ. **Figure S9.** Bayesian colocalization test of ACEIs with schizophrenia. Panel A: The posterior probability for the distinct causal variants model (PP_H3_) is 0.011. The posterior probability for the model with a shared causal variant (PP_H4_) is 0.98. Panel B: The posterior probability for the distinct causal variants model (PP_H3_) is 0.054. The posterior probability for the model with a shared causal variant (PP_H4_) is 0.35. **Table S1.** Information on genetic instruments. **Table S2.** Power calculation. power calculation for detectable odds ratio (per 5 mmHg lower in SBP) at 80% power. The R^2^ was calculated as beta^2^* 2 * MAF * (1-MAF), where beta is the SNP-outcome association standardized to the phenotypic variance and MAF is the minor allele frequency of the SNP. The R^2^ for ACEI among Europeans is 0.0015, and among the East Asians is 0.0016. The R^2^ for BB among the Europeans is 0.009, and among east Asians is 0.0018. The R^2^ for CCB among the Europeans is 0.030, and 0.046 among the East Asians. **Table S3.** Sensitivity analysis using eQTL SNPs to proxy antihypertensive drugs in the European population. **Table S4.** I^2^_GX_ of CCBs MR-Egger analysis. **Table S5.** Sensitivity analysis using GWAS of SBP in the UK Biobank without adjustment of BMI. **Table S6.** Sensitivity analysis for bipolar disorder GWAS without UK Biobank participants. **Table S7.** Sensitivity analysis using DBP-associated SNPs to proxy antihypertensive drugs in the European population.

## Data Availability

The datasets generated and/or analysed during the current study are available in the psychiatric genomics consortium repository, https://pgc.unc.edu/for-researchers/download-results/.

## Abbreviations

ACEIs: Angiotensin-converting-enzyme inhibitors
BBs: Beta blockers
BD: Bipolar disorder
BMI: Body mass index
CCBs: Calcium channel blockers
CI: Confidence interval
GWAS: Genome-wide association studies
IVW: Inverse variance weighting
LD: Linkage disequilibrium
MDD: Major depression disorder
MR: Mendelian randomization
MR-PRESSO: Mendelian randomization pleiotropy residual sum and outlier
NOME: NO Measurement Error
OR: Odds ratio
PGC: Psychiatric Genomics Consortium
RAS: Renin-angiotensin system
RCTs: Randomized controlled trials
SBP: Systolic blood pressure
SCZ: Schizophrenia
SNPs: Single-nucleotide polymorphisms
