# Supplementary material for: Genetic proxies for antihypertensive drugs and mental disorders: Mendelian randomization study in European and East Asian populations
Source: BMC Med. 2024 Jan 2;22:6. doi: 10.1186/s12916-023-03218-6 (PMC10763027; doi:10.1186/s12916-023-03218-6)
Supplement: Supplementary file 1 — Additional file 1: Supplementary Methods. Flow chart of instrument selection and study design. Figure S1. Associations of BBs and CCBs using SNPs selected from LD threshold at r2< 0.01 in the European population. Two SNPs were used for genetic proxies for BBs and 8 SNPs were used for genetic proxies for CCBs. Figure S2. MR analysis for CCBs excluding pleiotropic SNPs related to CACNA1C gene in the European population and excluding pleiotropic SNPs related to CACNAB2 gene in the East Asian population. 22 SNPs served as genetic instruments in the European population and 14 SNPs served as genetic instruments in the East Asian population. Figure S3. MR analysis for CCBs excluding pleiotropic SNPs related to BMI. After removing rs3821843 and rs10828399 for Europeans and rs61842677 for East Asians related to BMI, 22SNPs served as genetic instruments in the European population and 20 SNPs served as genetic instruments in the East Asian population. Figure S4. MR sensitivity analysis for CCBs excluding pleiotropic SNPs related to cause of death. After removing two SNPs (rs113210396 and rs72786098) related to cause of death for the European population, 22SNPs served as genetic instruments for CCBs. Figure S5. MR sensitivity analysis for CCBs excluding pleiotropic SNPs related to BMI, CACNA1C gene, and cause of death. After removing all six SNPs related to CACNA1C, BMI and cause of death for the European population, 18 SNPs served as genetic instruments for CCBs. Figure S6. Associations of CCBs using SNPs selected from LD threshold r2< 0.01 and excluded pleiotropic SNPs related to BMI and CACNA1C gene. After removing 2 SNPs related to risk genes and potential confounder BMI for the European population, 6 SNPs served as genetic instruments. Figure S7. MR analysis of gene-specific effects of CCBs. Nsnp (column 4) indicates the number of SNPs aggregated for each gene targeted region of CCBs. Figure S8. Visual representation of the MR-Egger estimates of the genetic associations of [file 12916_2023_3218_MOESM1_ESM.docx]

**For the paper titled “Genetic proxies for antihypertensive drugs and mental disorders: Mendelian randomization study in European and East Asian populations”**

Additional file

[Supplementary Methods: Flow chart of instrument selection and study design 2](#_Toc153397155)

[Figure S1: Associations of BBs and CCBs using SNPs selected from LD threshold at r^2^ < 0.01 in the European population 3](#_Toc153397156)

[Figure S2: MR analysis for CCBs excluding pleiotropic SNPs related to CACNA1C gene in the European population and excluding pleiotropic SNPs related to CACNAB2 gene in the East Asian population 4](#_Toc153397157)

[Figure S3: MR analysis for CCBs excluding pleiotropic SNPs related to BMI 5](#_Toc153397158)

[Figure S4: MR sensitivity analysis for CCBs excluding pleiotropic SNPs related to cause of death 6](#_Toc153397159)

[Figure S5: MR sensitivity analysis for CCBs excluding pleiotropic SNPs related to BMI, CACNA1C gene, and cause of death 7](#_Toc153397160)

[Figure S6: Associations of CCBs using SNPs selected from LD threshold r^2^ < 0.01 and excluded pleiotropic SNPs related to BMI and CACNA1C gene 8](#_Toc153397161)

[Figure S7: MR analysis of gene-specific effects of CCBs 9](#_Toc153397162)

[Figure S8: Visual representation of the MR-Egger estimates of the genetic associations of CCBs with BD and SCZ 10](#_Toc153397163)

[Figure S9: Bayesian colocalization test of ACEIs with schizophrenia 11](#_Toc153397164)

[Table S1: Information on genetic instruments 12](#_Toc153397165)

[Table S2: Power calculation 18](#_Toc153397166)

[Table S3: Sensitivity analysis using eQTL SNPs to proxy antihypertensive drugs in the European population 19](#_Toc153397167)

[Table S4: I^2^_GX_ of CCBs MR-Egger analysis 20](#_Toc153397168)

[Table S5: Sensitivity analysis using GWAS of SBP in the UK Biobank without adjustment of BMI 21](#_Toc153397169)

[Table S6: Sensitivity analysis for bipolar disorder GWAS without UK Biobank participants 22](#_Toc153397170)

[Table S7: Sensitivity analysis using DBP-associated SNPs to proxy antihypertensive drugs in the European population 23](#_Toc153397171)

# Supplementary Methods: Flow chart of instrument selection and study design

# Figure S1: Associations of BBs and CCBs using SNPs selected from LD threshold at r^2^ < 0.01 in the European population

1. BBs


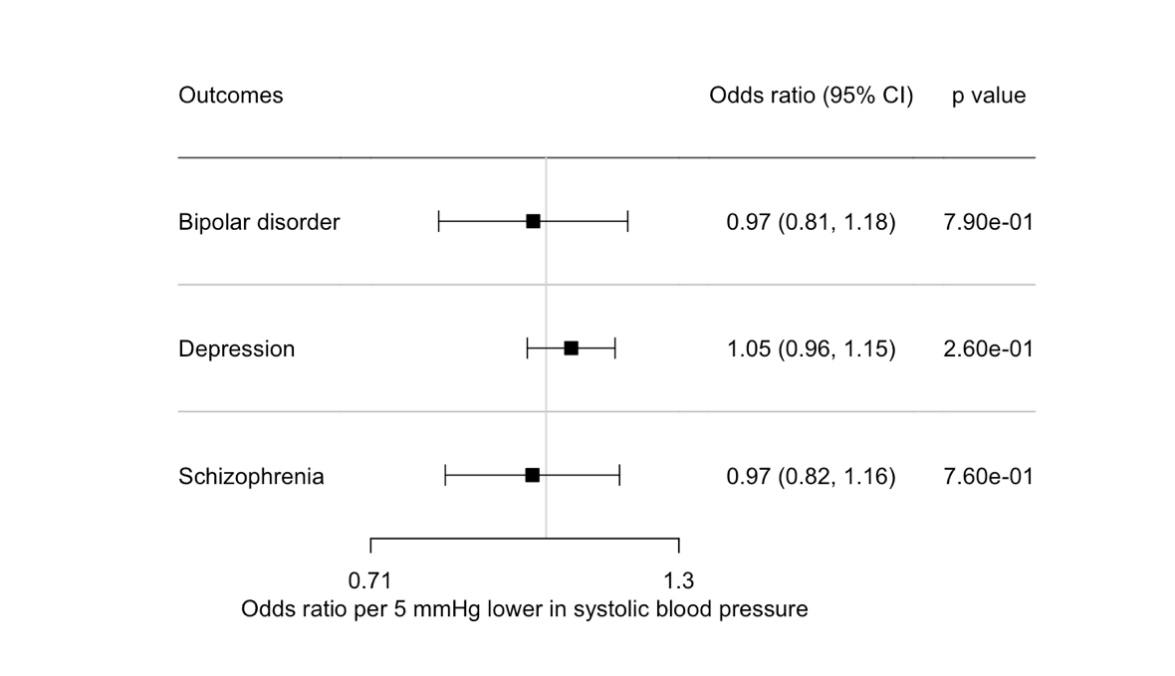


1. CCBs


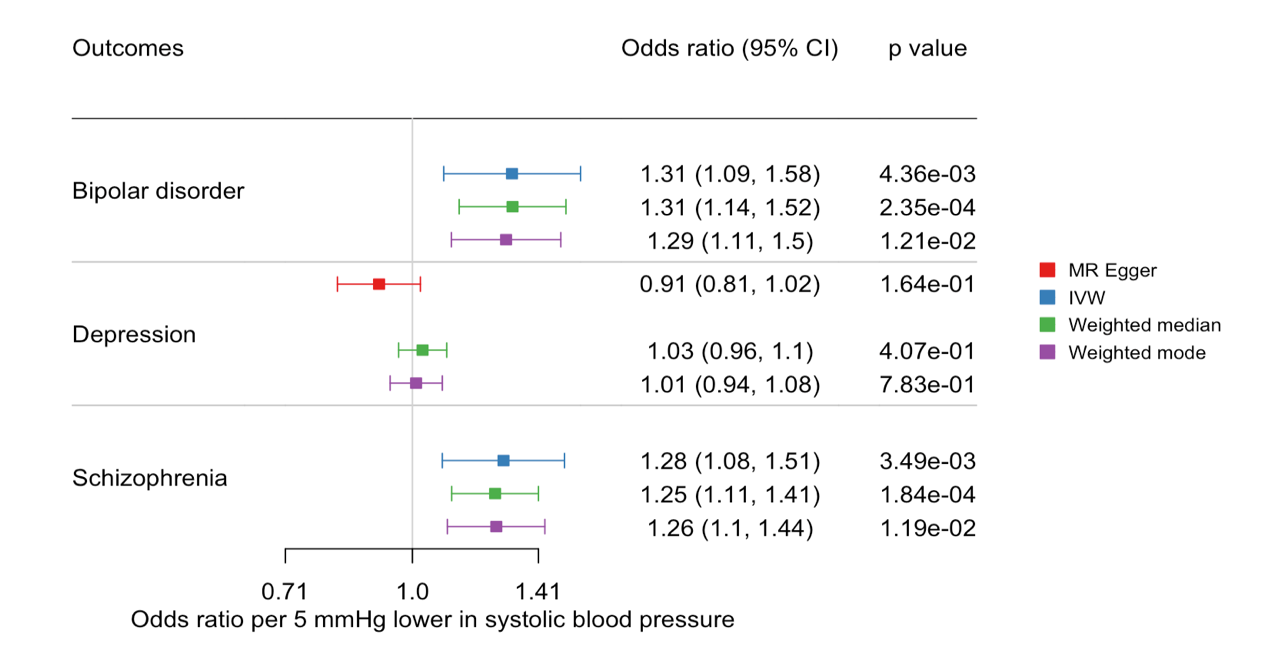


Footnote: 2 SNPs were used for BBs and 8 SNPs were used for CCBs

# Figure S2: MR analysis for CCBs excluding pleiotropic SNPs related to *CACNA1C* gene in the European population and excluding pleiotropic SNPs related to *CACNAB2* gene in the East Asian population


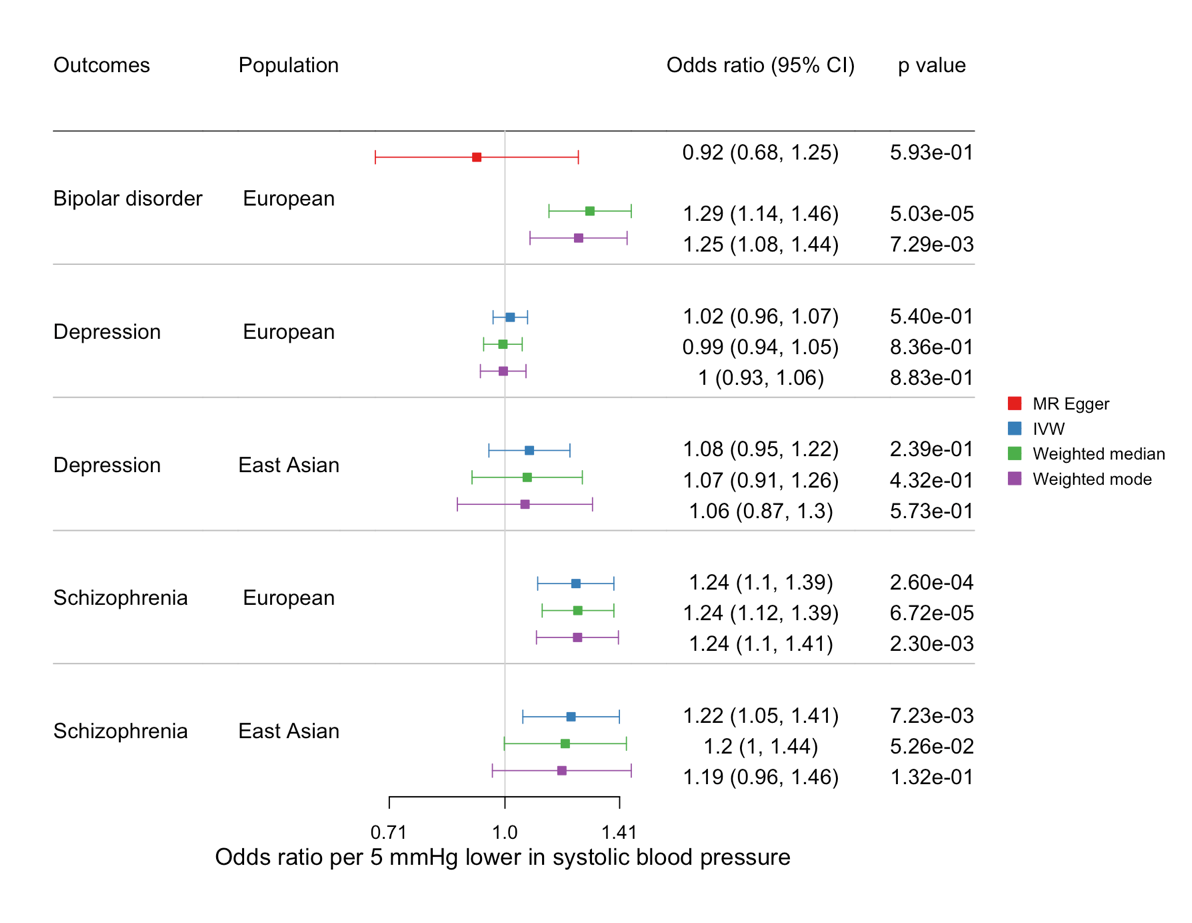


Footnote: 22 SNPs served as genetic instruments in the European population and 14 SNPs served as genetic instruments in the East Asian population

# Figure S3: MR analysis for CCBs excluding pleiotropic SNPs related to BMI


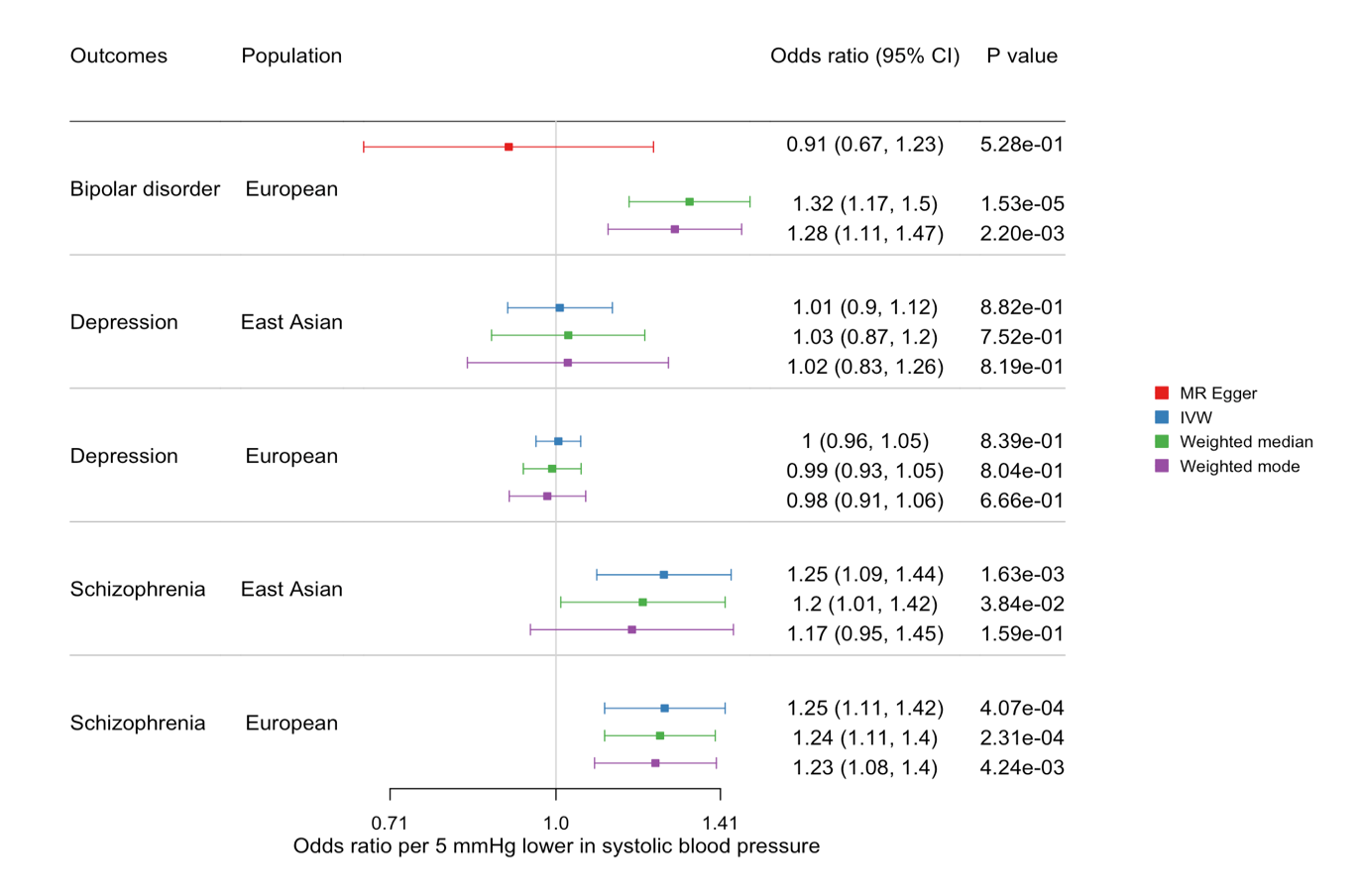


Footnote: after removing rs3821843 and rs10828399 for Europeans and rs61842677 for East Asians related to BMI, 22SNPs served as genetic instruments in the European population and 20 SNPs served as genetic instruments in the East Asian population

# Figure S4: MR sensitivity analysis for CCBs excluding pleiotropic SNPs related to cause of death


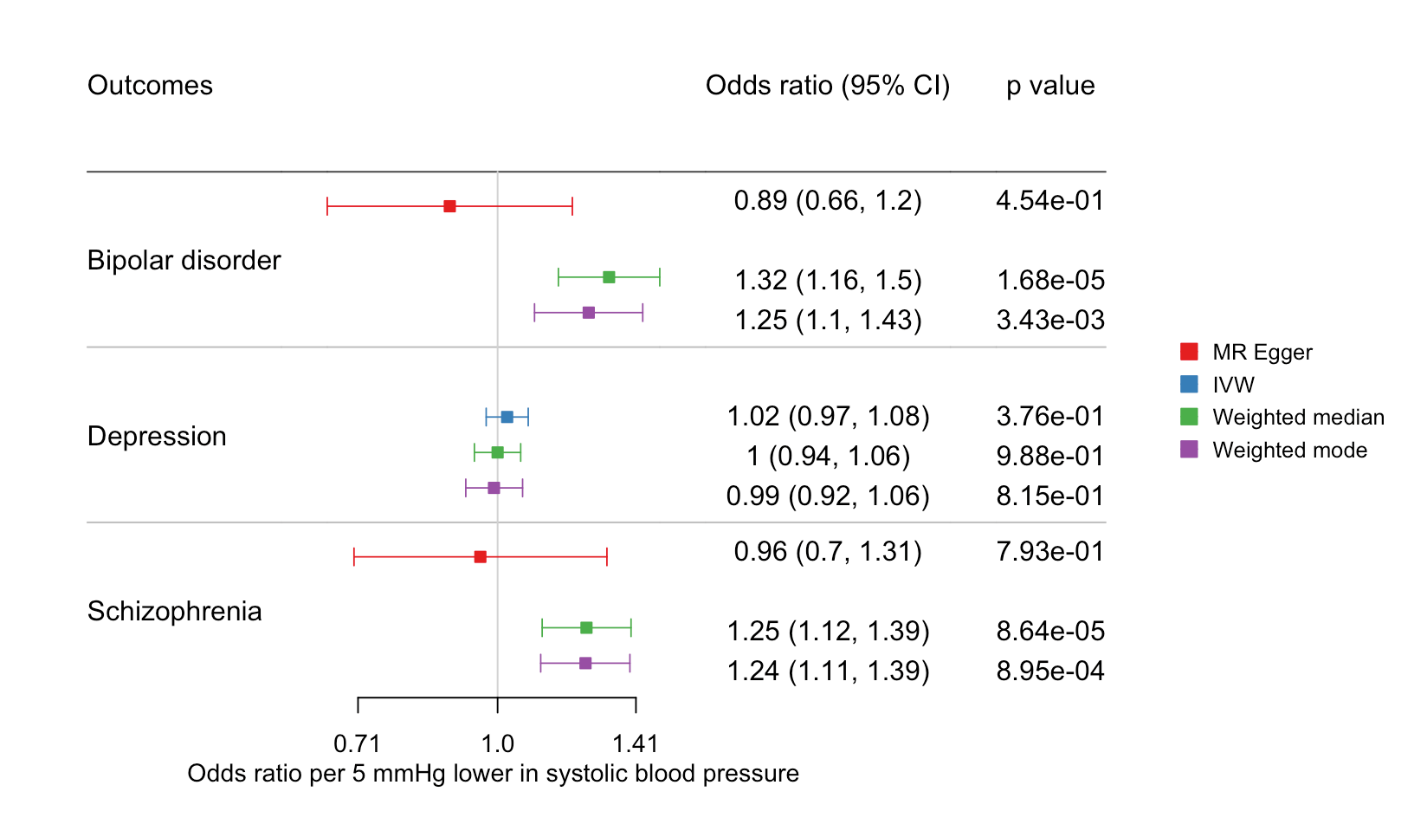


Footnote: after removing two SNPs (rs113210396 and rs72786098) related to cause of death for the European population, 22SNPs served as genetic instruments

# Figure S5: MR sensitivity analysis for CCBs excluding pleiotropic SNPs related to BMI, *CACNA1C* gene, and cause of death


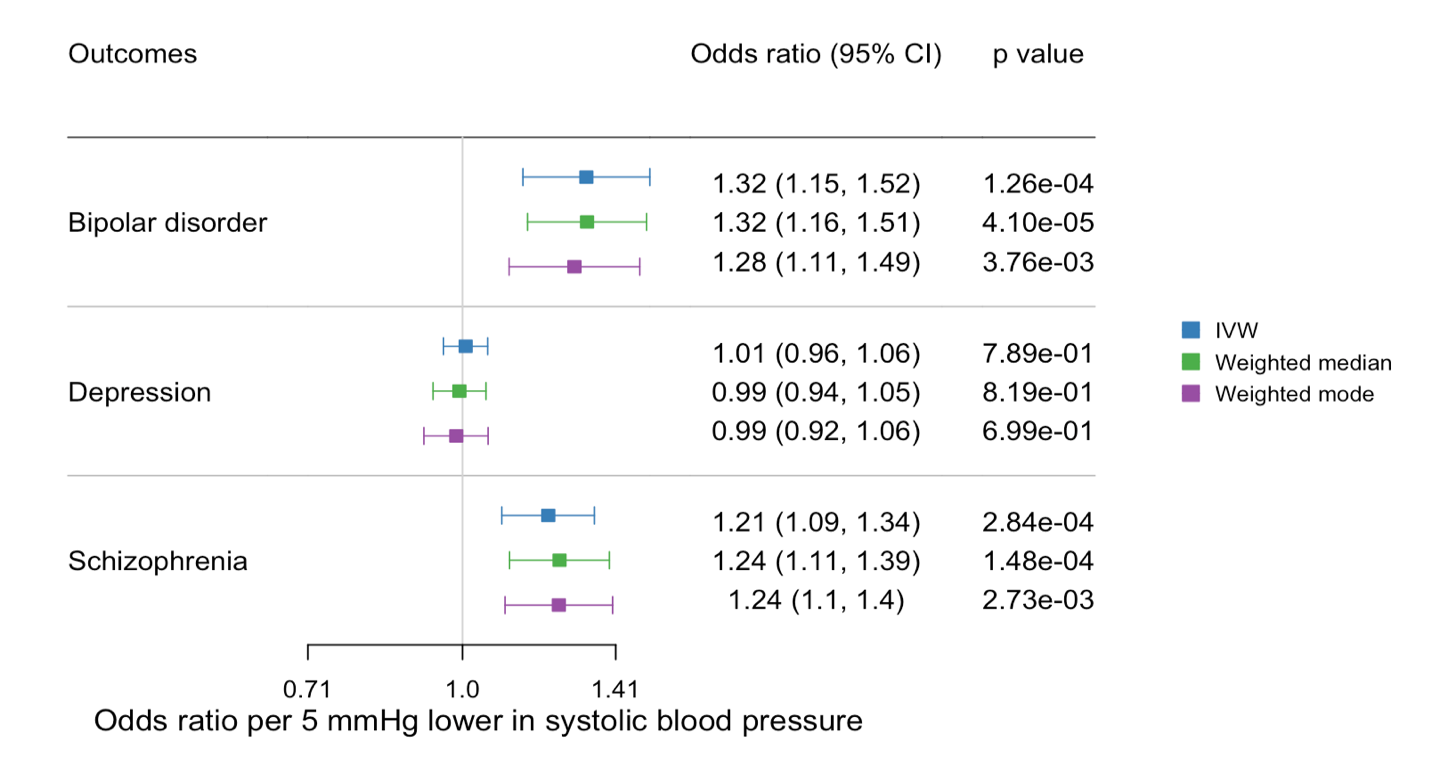


Footnote: after removing all six SNPs related to *CACNA1C*, BMI and cause of death for the European population, 18 SNPs served as genetic instruments

# Figure S6: Associations of CCBs using SNPs selected from LD threshold r^2^ < 0.01 and excluded pleiotropic SNPs related to BMI and *CACNA1C* gene


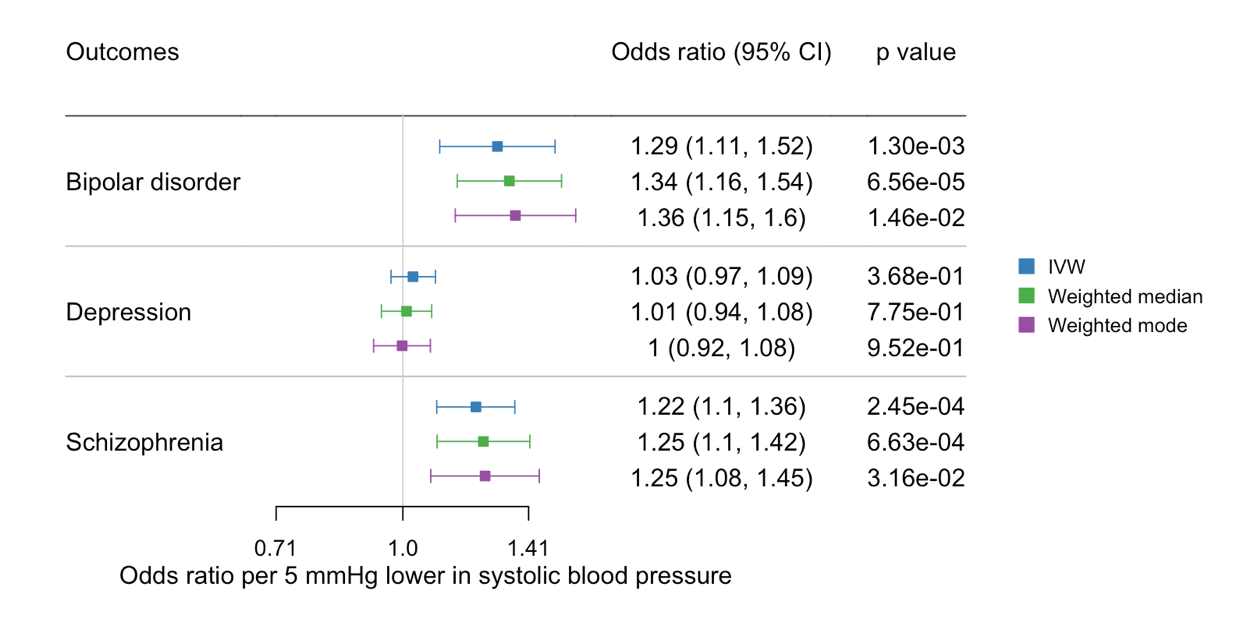


Footnote: after removing 2 SNPs related to risk genes and potential confounders BMI for the European population, 6 SNPs served as genetic instruments

# Figure S7: MR analysis of gene-specific effects of CCBs


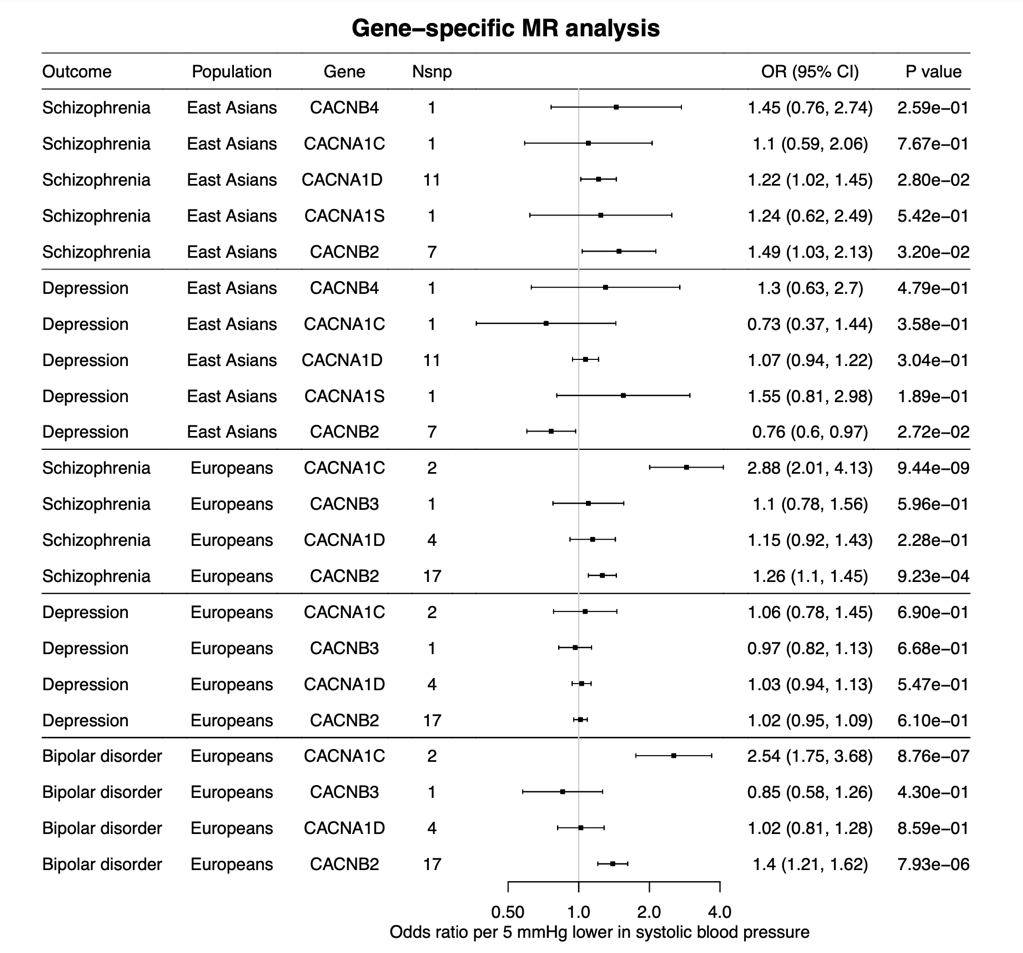


footnote:

Nsnp indicates the number of SNPs aggregated for each gene targeted region of CCBs.

# Figure S8: Visual representation of the MR-Egger estimates of the genetic associations of CCBs with BD and SCZ


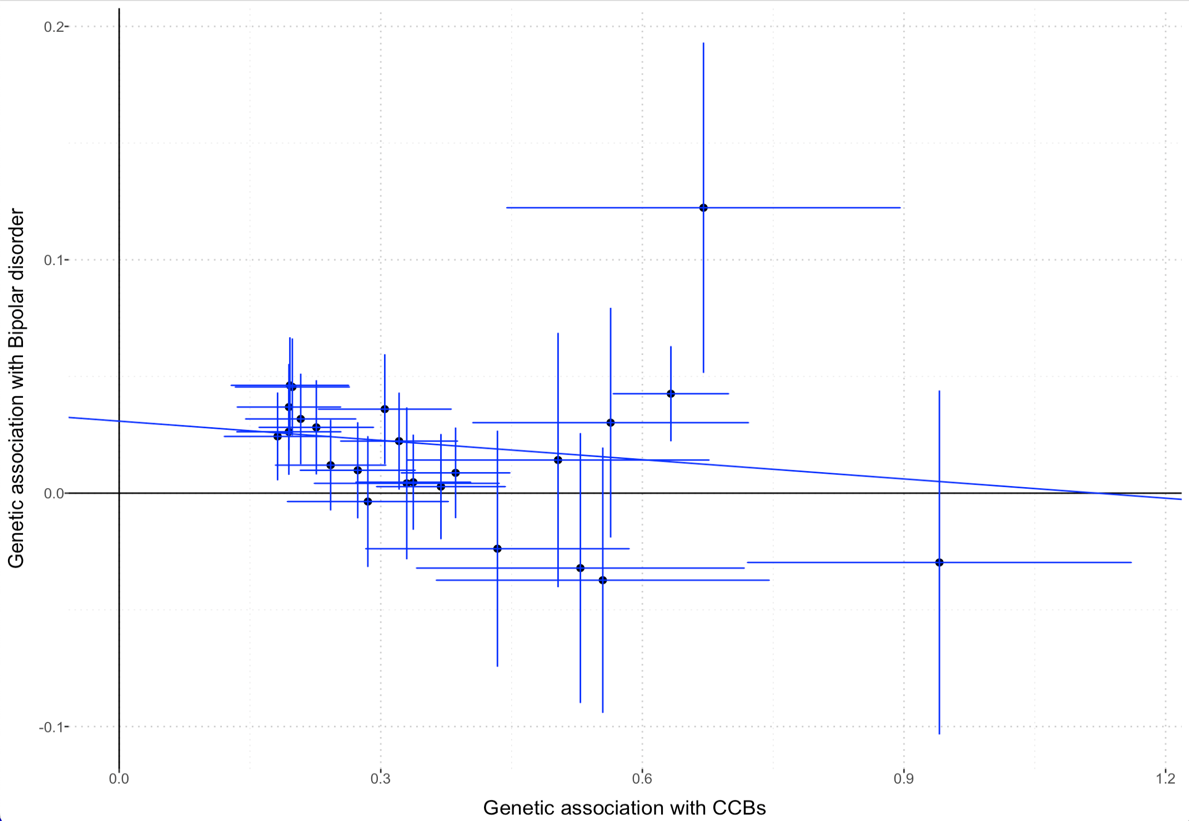


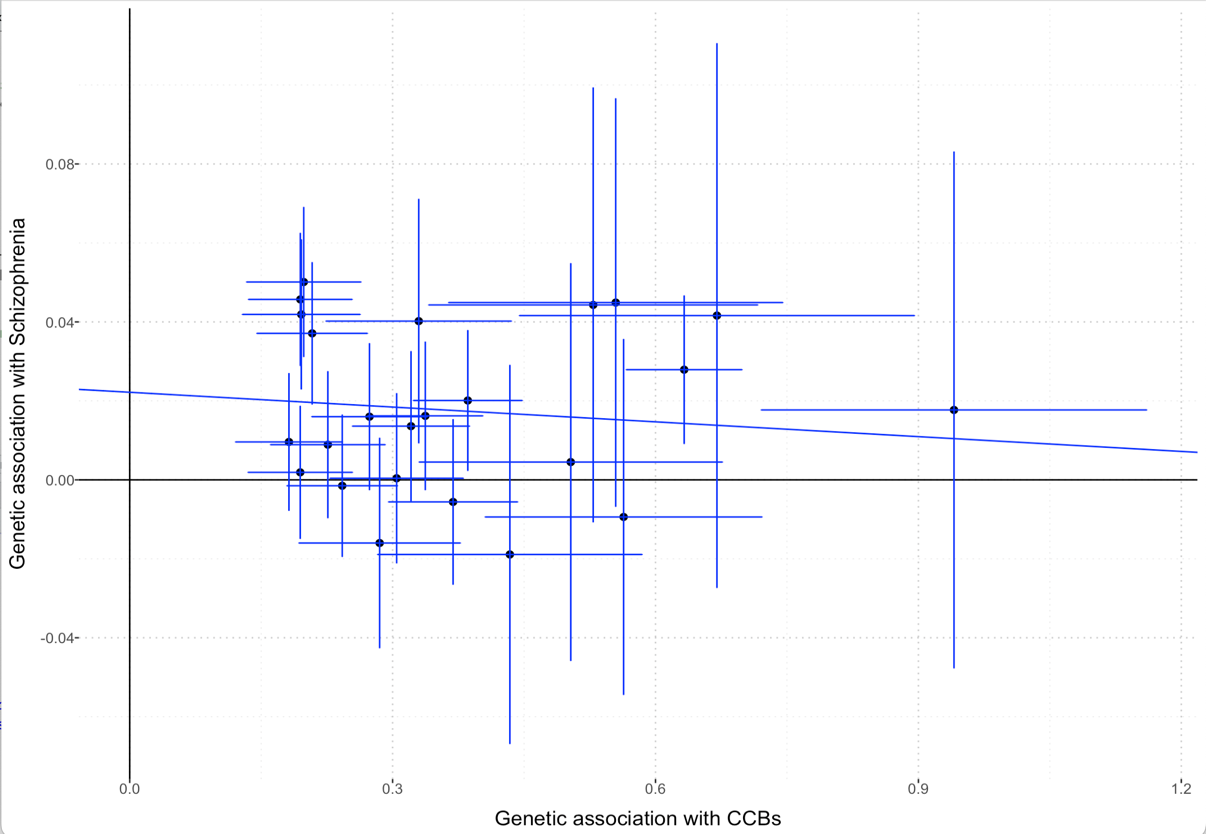


# Figure S9: Bayesian colocalization test of ACEIs with schizophrenia

1. **In Europeans**


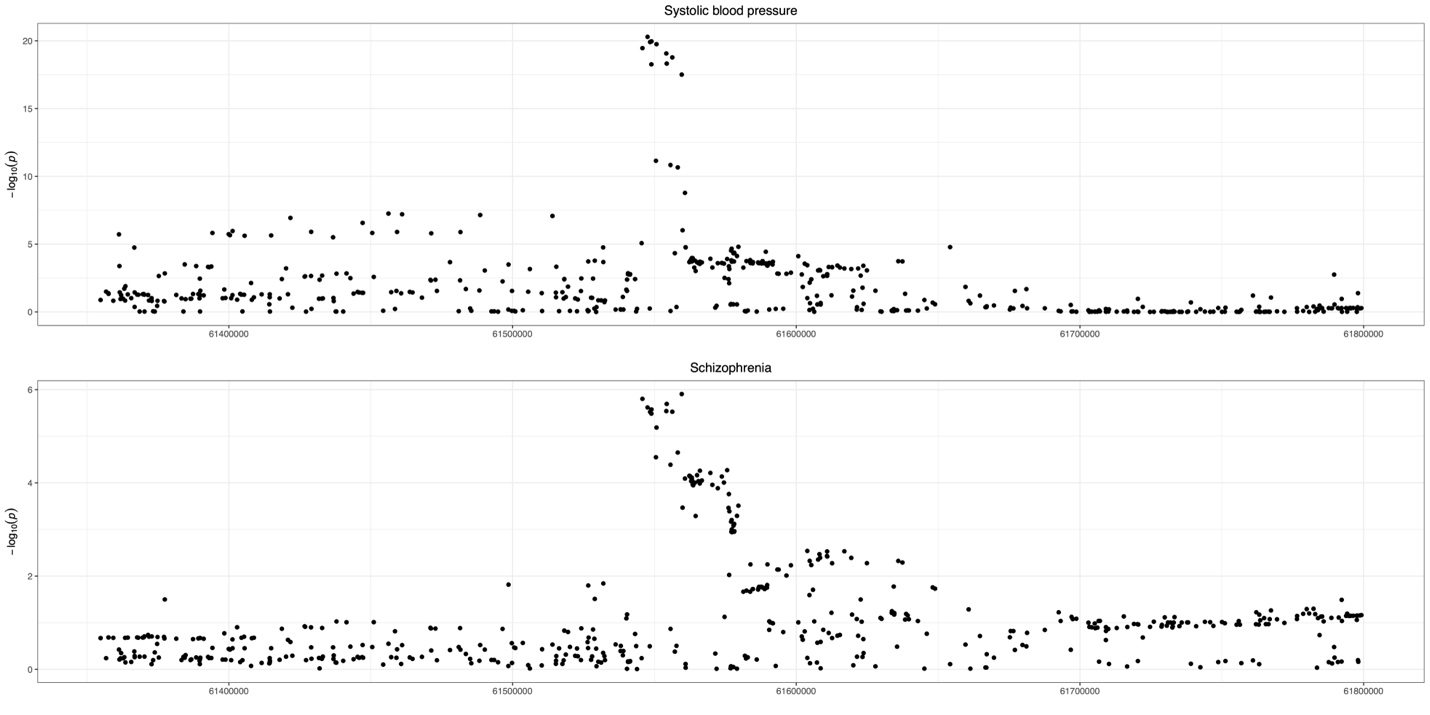


The posterior probability for the distinct causal variants model (PP_H3_), is 0.011.

The posterior probability for the model with a shared causal variant (PP_H4_) is 0.98.

1. **In East Asians**


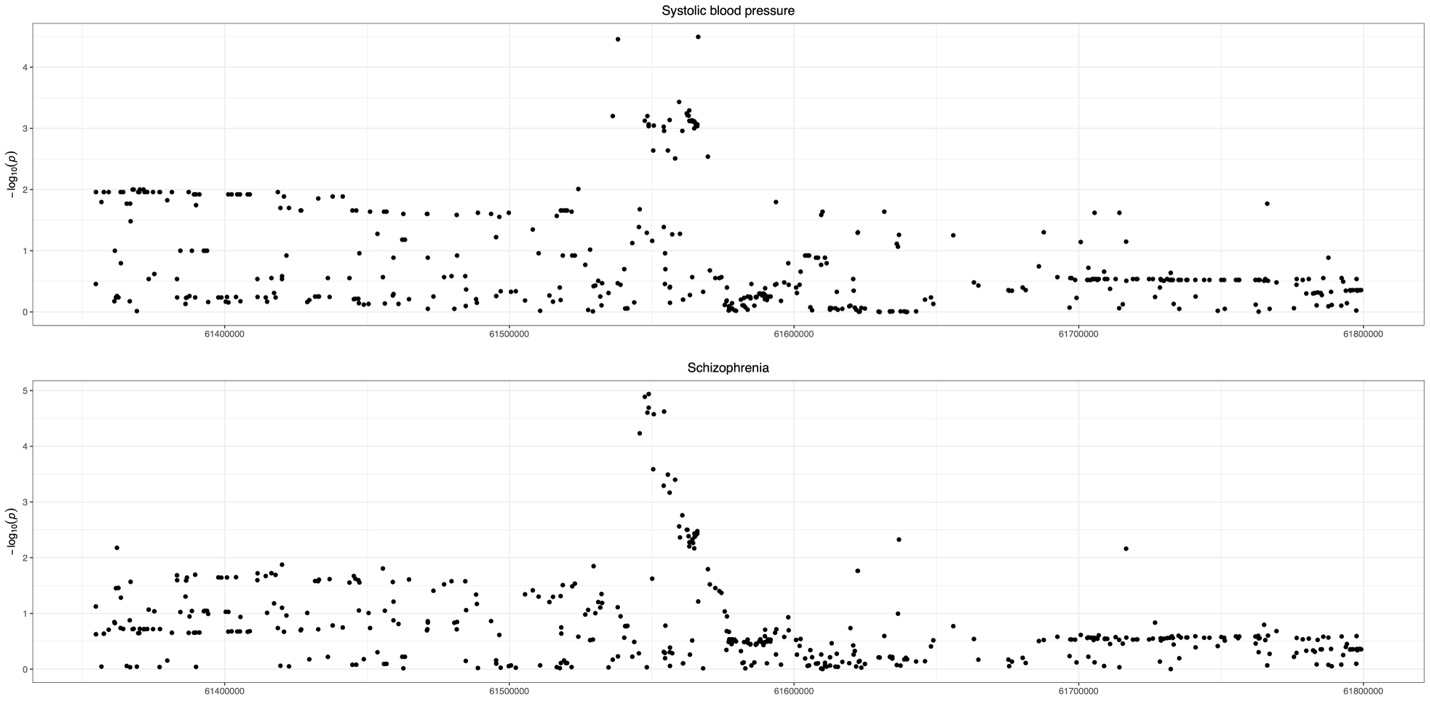


The posterior probability for the distinct causal variants model (PP_H3_) is 0.054.

The posterior probability for the model with a shared causal variant (PP_H4_) is 0.35.

# Table S1: Information on genetic instruments

1. **ACEIs**

**Genetic instruments for ACEIs used in the main analysis**

| Population | SNP | Chr | Position | Gene | EA | OA | EAF | Effect | SE | P |
| --- | --- | --- | --- | --- | --- | --- | --- | --- | --- | --- |
| European | rs4291 | 17 | 61554194 | *ACE* | A | T | 0.6155 | -0.2839 | 0.0312 | 8.65E-20 |
| East Asian | rs4308 | 17 | 61559625 | *ACE* | G | A | 0.6401 | -0.01501 | 0.003966 | 0.000154 |

**Genetic instruments for ACEIs identified based on eQTL**

| Gene | SNP | EA | EAF | Effect | SE | R2 | Fstat |
| --- | --- | --- | --- | --- | --- | --- | --- |
| *ACE* | rs4968783 | A | 0.62 | -0.01 | 0.002 | 4.04E-05 | 16 |

**Genetic instruments for ACEIs and genetic associations with diastolic blood pressure (DBP)**

| Gene | SNP | Chr | Position | EA | OA | EAF | Effect | SE | P | Fstat |
| --- | --- | --- | --- | --- | --- | --- | --- | --- | --- | --- |
| *ACE* | rs4291 | 17 | 61554194 | A | T | 0.6163 | -0.1785 | 0.018 | 3.7E-23 | 98 |

1. **BBs**

**Genetic instruments for BBs for Europeans used in primary analysis**

| Gene | SNP | Chr | Position | EA | OA | EAF | Effect | SE | P |
| --- | --- | --- | --- | --- | --- | --- | --- | --- | --- |
| *ADRB1* | rs11196549^*^ | 10 | 115707298 | A | G | 0.0425 | -0.6884 | 0.0784 | 1.58E-18 |
| *ADRB1* | rs11196597 | 10 | 115788094 | A | G | 0.133 | -0.2858 | 0.0458 | 4.23E-10 |
| *ADRB1* | rs17875473 | 10 | 115800294 | T | C | 0.0871 | -0.3283 | 0.0552 | 2.66E-09 |
| *ADRB1* | rs1801253^*^ | 10 | 115805056 | C | G | 0.7338 | -0.4626 | 0.0344 | 2.84E-41 |
| *ADRB1* | rs4359161 | 10 | 115826508 | A | G | 0.1812 | 0.2662 | 0.0391 | 9.46E-12 |
| *ADRB1* | rs460718 | 10 | 115721364 | A | G | 0.3266 | 0.2764 | 0.0324 | 1.36E-17 |

^*^these 2 SNPs were selected with LD of r2<0.01 in sensitivity analysis

**Genetic instruments for BBs for East Asians used in primary analysis**

| SNP | Chr | Position | EA | OA | EAF | Effect | SE | P |
| --- | --- | --- | --- | --- | --- | --- | --- | --- |
| rs10885532 | 10 | 115828256 | G | C | 0.2397 | -0.01614 | 0.00472 | 0.00062941 |
| rs180912 | 10 | 115741828 | G | T | 0.4221 | 0.01841 | 0.003949 | 3.14E-06 |
| rs78006240 | 10 | 115717317 | C | T | 0.123 | -0.02201 | 0.006031 | 0.0002627 |
| rs80113039 | 10 | 115824246 | A | G | 0.0116 | -0.06272 | 0.01885 | 0.00087921 |

**Genetic instruments for BBs identified based on eQTL**

| Gene | SNP | EA | EAF | Effect | SE | R2 | Fstat |
| --- | --- | --- | --- | --- | --- | --- | --- |
| *ADRA1B* | rs13159674 | G | 0.2 | 0.01 | 0.003 | 2.54E-05 | 10.1 |
| *ADRA1B* | rs17645325 | G | 0.13 | 0.02 | 0.004 | 6.90E-05 | 27.4 |
| *ADRA1A* | rs1899494 | A | 0.26 | 0.01 | 0.003 | 3.47E-05 | 13.8 |
| *ADRB3* | rs35866749 | G | 0.16 | -0.01 | 0.003 | 2.76E-05 | 10.9 |
| *ADRB1* | rs7076938 | A | 0.74 | 0.02 | 0.003 | 0.000185 | 73.5 |
| *ADRB1* | rs74717224 | A | 0.06 | -0.02 | 0.005 | 3.40E-05 | 13.5 |
| *ADRB2* | rs7737361 | A | 0.19 | -0.01 | 0.003 | 4.85E-05 | 19.2 |

**Genetic instruments for BBs and genetic associations with DBP**

| Gene | SNP | Chr | Position | EA | OA | EAF | Effect | SE | P | Fstat |
| --- | --- | --- | --- | --- | --- | --- | --- | --- | --- | --- |
| *ADRB1* | rs717368 | 10 | 115707296 | A | G | 0.9155 | -0.2035 | 0.0325 | 3.68E-10 | 39 |
| *ADRB1* | rs180944 | 10 | 115717357 | A | C | 0.3707 | -0.1092 | 0.018 | 1.30E-09 | 37 |
| *ADRB1* | rs11196595 | 10 | 115784376 | T | C | 0.869 | 0.1467 | 0.0257 | 1.17E-08 | 33 |
| *ADRB1* | rs72823013 | 10 | 115786233 | A | G | 0.1228 | 0.1679 | 0.0266 | 2.86E-10 | 40 |
| *ADRB1* | rs11196597 | 10 | 115788094 | A | G | 0.1329 | 0.1817 | 0.0262 | 3.82E-12 | 48 |
| *ADRB1* | rs17875473 | 10 | 115800294 | T | C | 0.0865 | 0.2459 | 0.0317 | 8.64E-15 | 60 |
| *ADRB1* | rs80113039 | 10 | 115824246 | A | G | 0.033 | -0.2973 | 0.0514 | 7.10E-09 | 33 |

1. **CCBs**

**Genetic instruments for CCBs used in primary analysis for the European population (r^2^ < 0.1)**

| Gene | SNP | Chr | Position | EA | OA | EAF | Effect | SE | P |
| --- | --- | --- | --- | --- | --- | --- | --- | --- | --- |
| *CACNA1D* | rs3821843^*^ | 3 | 53558012 | A | G | 0.6808 | 0.3373 | 0.0335 | 6.56E-24 |
| *CACNA1D* | rs114987861 | 3 | 53605712 | A | G | 0.0284 | 0.5289 | 0.0958 | 3.36E-08 |
| *CACNA1D* | rs113210396^*^ | 3 | 53612327 | T | G | 0.0451 | -0.4338 | 0.0770 | 1.76E-08 |
| *CACNA1D* | rs7340705 | 3 | 53734443 | T | C | 0.6732 | -0.2425 | 0.0322 | 4.87E-14 |
| *CACNB2* | rs2488136 | 10 | 18334521 | A | G | 0.2875 | 0.2261 | 0.0334 | 1.22E-11 |
| *CACNB2* | rs1888693 | 10 | 18440444 | A | G | 0.3449 | 0.3858 | 0.0317 | 4.69E-34 |
| *CACNB2* | rs16916914 | 10 | 18457722 | T | C | 0.9631 | -0.5636 | 0.0806 | 2.72E-12 |
| *CACNB2* | rs7076319 | 10 | 18459450 | A | G | 0.7339 | -0.3210 | 0.0341 | 5.07E-21 |
| *CACNB2* | rs61278674 | 10 | 18481737 | A | G | 0.9062 | -0.3298 | 0.0540 | 1.03E-09 |
| *CACNB2* | rs1779209 | 10 | 18514561 | T | C | 0.2876 | 0.2736 | 0.0336 | 4.23E-16 |
| *CACNB2* | rs10828399^*^ | 10 | 18553968 | A | G | 0.5218 | -0.1947 | 0.0302 | 1.10E-10 |
| *CACNB2* | rs10828452 | 10 | 18592450 | A | T | 0.7930 | 0.3046 | 0.0388 | 4.20E-15 |
| *CACNB2* | rs10828542 | 10 | 18627285 | A | G | 0.6137 | 0.1817 | 0.0311 | 5.18E-09 |
| *CACNB2* | rs12780039 | 10 | 18678987 | C | G | 0.1210 | 0.2852 | 0.0470 | 1.26E-09 |
| *CACNB2* | rs112133583 | 10 | 18695681 | T | C | 0.0299 | -0.5546 | 0.0973 | 1.18E-08 |
| *CACNB2* | rs11014170 | 10 | 18710991 | A | G | 0.0206 | -0.6701 | 0.1150 | 5.61E-09 |
| *CACNB2* | rs7923191 | 10 | 18727901 | A | G | 0.7918 | -0.3690 | 0.0376 | 1.10E-22 |
| *CACNB2* | rs12258967 | 10 | 18727959 | C | G | 0.7047 | 0.6327 | 0.0337 | 1.08E-78 |
| *CACNB2* | rs72786098^*^ | 10 | 18729855 | A | G | 0.0322 | -0.5033 | 0.0883 | 1.18E-08 |
| *CACNB2* | rs1998822 | 10 | 18755664 | A | G | 0.7234 | -0.1958 | 0.0343 | 1.15E-08 |
| *CACNB2* | rs4748474 | 10 | 18790727 | A | G | 0.5214 | 0.1946 | 0.0304 | 1.61E-10 |
| *CACNB3* | rs150857355 | 12 | 49209340 | C | G | 0.0217 | 0.9406 | 0.1122 | 5.20E-17 |
| *CACNA1C* | rs2239046^*^ | 12 | 2434419 | A | G | 0.6817 | 0.2082 | 0.0322 | 9.58E-11 |
| *CACNA1C* | rs714277^*^ | 12 | 2514270 | T | C | 0.2834 | 0.1986 | 0.0333 | 2.38E-09 |

^*^rs3821843 and rs10828399 are related to BMI; rs714277 and rs223046 correspond to *CACNA1C* gene and rs714277 is related to schizophrenia; rs113210396 and rs72786098 are related to cause of death

**Genetic instruments for CCBs used in primary analysis for the East Asian population**

| Gene | SNP | CHR | Position | EA | OA | EAF | Effect | SE | P |
| --- | --- | --- | --- | --- | --- | --- | --- | --- | --- |
| *CACNA1D* | rs9814480 | 3 | 53590465 | C | T | 0.1238 | 0.04209 | 0.005778 | 3.23E-13 |
| *CACNA1D* | rs2680650 | 3 | 53775974 | G | T | 0.3106 | -0.01432 | 0.004128 | 0.0005215 |
| *CACNB4* | rs59655785 | 2 | 152494218 | A | G | 0.4379 | -0.01448 | 0.004422 | 0.00106001 |
| *CACNA1D* | rs78021240 | 3 | 53888840 | A | G | 0.1341 | -0.0272 | 0.005904 | 4.07E-06 |
| *CACNB2* | rs17611696 | 10 | 18673100 | C | T | 0.094 | -0.02167 | 0.006508 | 0.000871 |
| *CACNB2* | rs2497820 | 10 | 18368849 | G | A | 0.516 | -0.01451 | 0.003811 | 0.0001402 |
| *CACNB2* | rs72786097 | 10 | 18728998 | T | G | 0.351 | -0.01539 | 0.004215 | 0.0002614 |
| *CACNA1D* | rs55828852 | 3 | 53573886 | C | G | 0.2165 | 0.02241 | 0.004608 | 1.16E-06 |
| *CACNA1D* | rs4687590 | 3 | 53861137 | A | G | 0.4647 | -0.01919 | 0.003862 | 6.72E-07 |
| *CACNB2* | rs80255112 | 10 | 18409845 | A | G | 0.1901 | -0.01569 | 0.00495 | 0.001521 |
| *CACNA1D* | rs62250902 | 3 | 53842251 | C | T | 0.1591 | -0.0278 | 0.005264 | 1.28E-07 |
| *CACNA1S* | rs12749395 | 1 | 201131374 | C | T | 0.1563 | -0.017 | 0.005333 | 0.00143599 |
| *CACNB2* | rs117222257 | 10 | 18361003 | C | A | 0.1577 | -0.01675 | 0.005227 | 0.00134899 |
| *CACNA1D* | rs56021416 | 3 | 53594413 | A | C | 0.2809 | 0.02453 | 0.004262 | 8.59E-09 |
| *CACNA1D* | rs79370913 | 3 | 53742828 | G | A | 0.055 | -0.02667 | 0.008423 | 0.00154501 |
| *CACNA1D* | rs2358619 | 3 | 53343936 | T | C | 0.465 | 0.01492 | 0.003834 | 9.97E-05 |
| *CACNA1D* | rs3821851 | 3 | 53633122 | C | T | 0.3626 | 0.01862 | 0.003941 | 2.32E-06 |
| *CACNA1C* | rs7967032 | 12 | 2046099 | T | G | 0.6894 | -0.01607 | 0.004845 | 0.0009139 |
| *CACNB2* | rs61842677^*^ | 10 | 18597598 | G | A | 0.1656 | -0.0168 | 0.005104 | 0.0009969 |
| *CACNA1D* | rs74601753 | 3 | 53473900 | C | G | 0.0905 | 0.03651 | 0.007738 | 2.38E-06 |
| *CACNB2* | rs11013860 | 10 | 18654027 | A | C | 0.4728 | 0.01533 | 0.003792 | 5.29E-05 |

^*^ rs61842677 is related to BMI

**Genetic instruments for CCBs selected with LD of r^2^ < 0.01**

| Gene | SNP | Chr | Position | EA | OA | EAF | Effect | SE | P |
| --- | --- | --- | --- | --- | --- | --- | --- | --- | --- |
| *CACNB2* | rs2488136 | 10 | 18334521 | A | G | 0.7088 | -0.2261 | 0.0334 | 1.22E-11 |
| *CACNB2* | rs1888693 | 10 | 18440444 | G | A | 0.3425 | 0.3858 | 0.0317 | 4.69E-34 |
| *CACNB2* | rs79253631 | 10 | 18694223 | A | G | 0.01332 | 0.7774 | 0.1392 | 2.32E-08 |
| *CACNB2* | rs12258967 | 10 | 18727959 | C | G | 0.2979 | -0.6327 | 0.0337 | 1.08E-78 |
| *CACNA1C* | rs714277^*^ | 12 | 2514270 | C | T | 0.2838 | 0.1986 | 0.0333 | 2.38E-09 |
| *CACNB3* | rs150857355 | 12 | 49209340 | G | C | 0.02204 | 0.9406 | 0.1122 | 5.20E-17 |
| *CACNA1D* | rs3821843^*^ | 3 | 53558012 | G | A | 0.6786 | 0.3373 | 0.0335 | 6.56E-24 |
| *CACNA1D* | rs7340705 | 3 | 53734443 | T | C | 0.3224 | 0.2425 | 0.0322 | 4.87E-14 |

^*^ rs3821843 is related to BMI; rs714277 is related to *CACNA1C* gene and schizophrenia risk

**Genetic instruments for CCBs identified based on eQTL**

| Gene | SNP | EA | EAF | Effect | SE | R2 | Fstat |
| --- | --- | --- | --- | --- | --- | --- | --- |
| *CACNA1C* | rs10848645 | A | 0.55 | 0.01 | 0.002 | 4.47E-05 | 17.7 |
| *CACNA1D* | rs6445583 | A | 0.75 | 0.02 | 0.003 | 9.21E-05 | 36.5 |
| *CACNA1D* | rs79020595 | C | 0.02 | -0.04 | 0.01 | 4.35E-05 | 17.3 |
| *CACNA1G* | rs9890200 | C | 0.37 | -0.01 | 0.002 | 6.05E-05 | 24 |
| *CACNA1H* | rs117177120 | A | 0.06 | 0.02 | 0.005 | 5.64E-05 | 22.4 |
| *CACNA2D2* | rs34484573 | A | 0.13 | -0.02 | 0.004 | 0.000124 | 49.2 |
| *CACNB2* | rs10764322 | G | 0.31 | 0.02 | 0.003 | 0.000117 | 46.6 |
| *CACNB2* | rs7922241 | G | 0.79 | 0.01 | 0.003 | 5.41E-05 | 21.5 |
| *CACNB3* | rs12317778 | C | 0.08 | -0.02 | 0.004 | 7.88E-05 | 31.3 |

**Genetic instruments for CCBs and genetic associations with DBP**

| Gene | SNP | Chr | Position | EA | OA | EAF | Effect | SE | P | Fstat |
| --- | --- | --- | --- | --- | --- | --- | --- | --- | --- | --- |
| CACNA1C | rs4765675 | 12 | 2469497 | T | G | 0.6111 | -0.1026 | 0.0181 | 1.45E-08 | 32 |
| CACNA1C | rs7314860 | 12 | 2177546 | A | G | 0.1732 | -0.1358 | 0.0236 | 8.50E-09 | 33 |
| CACNA1C | rs7314860 | 12 | 2177546 | A | G | 0.1732 | -0.1358 | 0.0236 | 8.50E-09 | 33 |
| CACNA1D | rs113210396 | 3 | 53612327 | T | G | 0.0455 | -0.2539 | 0.0439 | 7.09E-09 | 33 |
| CACNA1D | rs11720002 | 3 | 53709642 | T | C | 0.724 | -0.1494 | 0.0193 | 1.12E-14 | 60 |
| CACNA1D | rs35593046 | 3 | 53553923 | T | G | 0.267 | -0.152 | 0.0201 | 4.35E-14 | 57 |
| CACNA1D | rs35593046 | 3 | 53553923 | T | G | 0.267 | -0.152 | 0.0201 | 4.35E-14 | 57 |
| CACNA1D | rs3774468 | 3 | 53629706 | T | G | 0.6162 | -0.1081 | 0.0181 | 2.23E-09 | 36 |
| CACNA1D | rs62251864 | 3 | 53559907 | A | G | 0.767 | -0.1159 | 0.0205 | 1.58E-08 | 32 |
| CACNA1D | rs62251864 | 3 | 53559907 | A | G | 0.767 | -0.1159 | 0.0205 | 1.58E-08 | 32 |
| CACNA2D2 | rs2236953 | 3 | 50424182 | T | G | 0.1338 | 0.1467 | 0.0255 | 8.71E-09 | 33 |
| CACNB2 | rs10764459 | 10 | 18680670 | A | G | 0.6372 | -0.1189 | 0.0181 | 5.15E-11 | 43 |
| CACNB2 | rs10828270 | 10 | 18439035 | A | G | 0.7793 | -0.1157 | 0.0209 | 3.32E-08 | 31 |
| CACNB2 | rs10828270 | 10 | 18439035 | A | G | 0.7793 | -0.1157 | 0.0209 | 3.32E-08 | 31 |
| CACNB2 | rs11013438 | 10 | 18590865 | A | C | 0.1668 | -0.1572 | 0.0244 | 1.24E-10 | 42 |
| CACNB2 | rs11014012 | 10 | 18681659 | T | G | 0.4686 | -0.1826 | 0.0175 | 1.81E-25 | 109 |
| CACNB2 | rs11014170 | 10 | 18710991 | A | G | 0.0206 | -0.3648 | 0.0657 | 2.82E-08 | 31 |
| CACNB2 | rs112133583 | 10 | 18695681 | T | C | 0.0303 | -0.3277 | 0.0552 | 2.88E-09 | 35 |
| CACNB2 | rs12241085 | 10 | 18538669 | C | G | 0.5067 | 0.1019 | 0.0175 | 6.15E-09 | 34 |
| CACNB2 | rs12416052 | 10 | 18789267 | T | C | 0.5941 | 0.1176 | 0.0178 | 4.14E-11 | 44 |
| CACNB2 | rs1277767 | 10 | 18498398 | A | G | 0.7783 | -0.1375 | 0.021 | 5.89E-11 | 43 |
| CACNB2 | rs138094231 | 10 | 18429624 | A | C | 0.0384 | 0.3514 | 0.0459 | 2.00E-14 | 59 |
| CACNB2 | rs138094231 | 10 | 18429624 | A | C | 0.0384 | 0.3514 | 0.0459 | 2.00E-14 | 59 |
| CACNB2 | rs17610275 | 10 | 18621630 | T | G | 0.9268 | 0.2887 | 0.0351 | 2.07E-16 | 68 |
| CACNB2 | rs2488136 | 10 | 18334521 | A | G | 0.2882 | 0.1061 | 0.0191 | 2.87E-08 | 31 |
| CACNB2 | rs34606998 | 10 | 18430855 | T | C | 0.2398 | 0.1556 | 0.0204 | 2.69E-14 | 58 |
| CACNB2 | rs34606998 | 10 | 18430855 | T | C | 0.2398 | 0.1556 | 0.0204 | 2.69E-14 | 58 |
| CACNB2 | rs4748444 | 10 | 18494482 | T | C | 0.6633 | 0.1284 | 0.0187 | 7.17E-12 | 47 |
| CACNB2 | rs61278674 | 10 | 18481737 | A | G | 0.9055 | -0.2193 | 0.0309 | 1.19E-12 | 50 |
| CACNB2 | rs6482385 | 10 | 18688883 | T | C | 0.5115 | 0.195 | 0.0174 | 4.03E-29 | 126 |
| CACNB2 | rs7076319 | 10 | 18459450 | A | G | 0.7346 | -0.1811 | 0.0196 | 2.35E-20 | 85 |
| CACNB2 | rs72786098 | 10 | 18729855 | A | G | 0.0325 | -0.3338 | 0.0502 | 3.05E-11 | 44 |
| CACNB2 | rs72786098 | 10 | 18729855 | A | G | 0.0325 | -0.3338 | 0.0502 | 3.05E-11 | 44 |
| CACNB2 | rs79666207 | 10 | 18583840 | T | C | 0.9823 | 0.3968 | 0.0694 | 1.10E-08 | 33 |
| CACNB2 | rs79666207 | 10 | 18583840 | T | C | 0.9823 | 0.3968 | 0.0694 | 1.10E-08 | 33 |
| CACNB3 | rs150857355 | 12 | 49209340 | C | G | 0.0217 | 0.4102 | 0.0643 | 1.79E-10 | 41 |
| CACNB3 | rs150857355 | 12 | 49209340 | C | G | 0.0217 | 0.4102 | 0.0643 | 1.79E-10 | 41 |
| SLC12A3 | rs10221121 | 16 | 56840328 | A | G | 0.2834 | -0.1082 | 0.0193 | 2.01E-08 | 31 |

# Table S2: Power calculation

| Exposure | Population | Traits | N_cases_ | N_controls_ | Detectable Odds ratio |
| --- | --- | --- | --- | --- | --- |
| ACEIs | Europeans | Bipolar disorder | 41,917 | 371,549 | (0.64, 1.57) |
|  | Europeans | Major depressive disorder | 170,756 | 329,443 | (0.77, 1.3) |
|  | Europeans | Schizophrenia | 53,386 | 77,258 | (0.61, 1.64) |
|  | East Asians | Major depressive disorder | 15,771 | 178,777 | (0.49, 2.03) |
|  | East Asians | Schizophrenia | 22,778 | 35,362 | (0.48, 2.06) |
| BBs | Europeans | Bipolar disorder | 41,917 | 371,549 | (0.81, 1.23) |
|  | Europeans | Major depressive disorder | 170,756 | 329,443 | (0.9, 1.12) |
|  | Europeans | Schizophrenia | 53,386 | 77,258 | (0.81, 1.23) |
|  | East Asians | Major depressive disorder | 15,771 | 178,777 | (0.71, 1.4) |
|  | East Asians | Schizophrenia | 22,778 | 35,362 | (0.71, 1.41) |
| CCBs | Europeans | Bipolar disorder | 41,917 | 371,549 | (0.9, 1.11) |
|  | Europeans | Major depressive disorder | 170,756 | 329,443 | (0.94, 1.06) |
|  | Europeans | Schizophrenia | 53,386 | 77,258 | (0.9, 1.12) |
|  | East Asians | Major depressive disorder | 15,771 | 178,777 | (0.88, 1.14) |
|  | East Asians | Schizophrenia | 22,778 | 35,362 | (0.87, 1.15) |

Footnote: power calculation for detectable odds ratio (per 5 mmHg lower in SBP) at 80% power. The R^2^ was calculated as beta^2^ * 2 * MAF * (1-MAF), where beta is the SNP-outcome association standardized to the phenotypic variance and MAF is the minor allele frequency of the SNP [1]. The R^2^ for ACEI among Europeans is 0.0015, and among the East Asians is 0.0016. The R^2^ for BB among the Europeans is 0.009, and among east Asians is 0.0018. The R^2^ for CCB among the Europeans is 0.030, and 0.046 among the East Asians.

**Reference**

1. Freeman G, Cowling BJ, Schooling CM: Power and sample size calculations for Mendelian randomization studies using one genetic instrument. Int J Epidemiol 2013, 42(4):1157-1163.

# Table S3: Sensitivity analysis using eQTL SNPs to proxy antihypertensive drugs in the European population

| Drug | Outcomes | No. of SNPs | Methods | OR, 95% CI | P value |
| --- | --- | --- | --- | --- | --- |
| ACEIs | Bipolar disorder | 1 | Wald ratio | 1.67 (1.02, 2.73) | 4.22E-02 |
|  | Depression | 1 | Wald ratio | 0.72 (0.57, 0.91) | 5.11E-03 |
|  | Schizophrenia | 1 | Wald ratio | 2.85 (1.80, 4.49) | 7.18E-06 |
| BBs | Bipolar disorder | 7 | IVW | 0.97 (0.82, 1.15) | 7.48E-01 |
|  | Bipolar disorder | 7 | Weighted median | 0.97 (0.78, 1.19) | 7.49E-01 |
|  | Bipolar disorder | 7 | Weighted mode | 1.01 (0.80, 1.29) | 9.06E-01 |
|  | Depression | 7 | IVW | 1.07 (0.97, 1.19) | 1.80E-01 |
|  | Depression | 7 | Weighted median | 1.11 (0.99, 1.25) | 6.97E-02 |
|  | Depression | 7 | Weighted mode | 1.14 (0.98, 1.32) | 1.00E-01 |
|  | Schizophrenia | 7 | IVW | 1.08 (0.92, 1.26) | 3.40E-01 |
|  | Schizophrenia | 7 | Weighted median | 1.08 (0.89, 1.31) | 4.27E-01 |
|  | Schizophrenia | 7 | Weighted mode | 1.08 (0.87, 1.35) | 4.87E-01 |
| CCBs | Bipolar disorder | 9 | IVW | 1.26 (0.99, 1.59) | 5.67E-02 |
|  | Bipolar disorder | 9 | Weighted median | 1.17 (0.98, 1.41) | 8.60E-02 |
|  | Bipolar disorder | 9 | Weighted mode | 1.15 (0.96, 1.39) | 1.26E-01 |
|  | Bipolar disorder | 9 | MR-Egger | 0.78 (0.42, 1.45) | 4.38E-01 |
|  | Bipolar disorder | 9 | MRPRESSO  Outlier-corrected | 1.17 (0.98, 1.40) | 1.24E-01 |
|  | Depression | 7 | IVW | 1.04 (0.96, 1.12) | 3.81E-01 |
|  | Depression | 7 | Weighted median | 1.08 (0.99, 1.18) | 9.78E-02 |
|  | Depression | 7 | Weighted mode | 1.10 (0.99, 1.23) | 7.65E-02 |
|  | Depression | 7 | MR-Egger | 1.02 (0.78, 1.33) | 8.89E-01 |
|  | Depression | 7 | MRPRESSO  (no outlier) | 1.04 (0.96, 1.12) | 4.15E-01 |
|  | Schizophrenia | 9 | IVW | 1.21 (0.92, 1.59) | 1.79E-01 |
|  | Schizophrenia | 9 | Weighted median | 1.15 (0.96, 1.37) | 1.31E-01 |
|  | Schizophrenia | 9 | Weighted mode | 1.11 (0.93, 1.32) | 2.53E-01 |
|  | Schizophrenia | 9 | MR-Egger | 0.60 (0.31, 1.16) | 1.30E-01 |
|  | Schizophrenia | 9 | MRPRESSO  Outlier-corrected | 1.20 (0.97, 1.48) | 1.38E-01 |

# Table S4: *I^2^_GX_* of CCBs MR-Egger analysis

| MR Analysis | Outcomes | *I^2^_GX_* for weighted MR-Egger |
| --- | --- | --- |
| Main analysis (24 SNPs) | bipolar disorder | 0.994 |
| Main analysis (24 SNPs) | schizophrenia | 0.999 |
| Sensitivity analysis (8 SNPs) | depression | 0.999 |
| Sensitivity analysis excluding pleiotropic SNPs related to *CACNA1C* gene (22 SNPs) | bipolar disorder | 0.995 |
| Sensitivity analysis excluding pleiotropic SNPs related to BMI (22 SNPs) | bipolar disorder | 0.993 |
| Sensitivity analysis excluding pleiotropic SNPs related to cause of death (22 SNPs) | bipolar disorder | 0.994 |
| Sensitivity analysis excluding pleiotropic SNPs related to cause of death (22 SNPs) | schizophrenia | 0.998 |

# Table S5: Sensitivity analysis using GWAS of SBP in the UK Biobank without adjustment of BMI

| Drugs | Outcomes | No of SNPs | Methods | OR, 95% CI | P value |
| --- | --- | --- | --- | --- | --- |
| ACEIs | Bipolar disorder | 1 | Wald ratio | 1.75 (1.03, 2.96) | 3.73E-02 |
|  | Depression | 1 | Wald ratio | 0.72 (0.56, 0.91) | 7.17E-03 |
|  | Schizophrenia | 1 | Wald ratio | 3.22 (1.97, 5.25) | 2.75E-06 |
| BBs | Bipolar disorder | 6 | IVW | 1.09 (0.93, 1.29) | 2.92E-01 |
|  | Bipolar disorder | 6 | Weighted median | 1.10 (0.90, 1.35) | 3.59E-01 |
|  | Bipolar disorder | 6 | Weighted mode | 1.10 (0.87, 1.39) | 4.29E-01 |
|  | Depression | 6 | IVW | 1.06 (0.98, 1.14) | 1.40E-01 |
|  | Depression | 6 | Weighted median | 1.04 (0.95, 1.15) | 3.86E-01 |
|  | Depression | 6 | Weighted mode | 1.01 (0.90, 1.13) | 9.00E-01 |
|  | Schizophrenia | 6 | IVW | 0.96 (0.78, 1.17) | 6.58E-01 |
|  | Schizophrenia | 6 | Weighted median | 1.04 (0.85, 1.29) | 6.86E-01 |
|  | Schizophrenia | 6 | Weighted mode | 1.04 (0.79, 1.37) | 7.95E-01 |
| CCBs | Bipolar disorder | 21 | IVW | 1.37 (1.17, 1.59) | 5.37E-05 |
|  | Bipolar disorder | 21 | Weighted median | 1.42 (1.23, 1.63) | 9.15E-07 |
|  | Bipolar disorder | 21 | Weighted mode | 1.32 (1.13, 1.53) | 3.39E-04 |
|  | Bipolar disorder | 21 | MR-Egger | 0.87 (0.62, 1.21) | 4.07E-01 |
|  | Bipolar disorder | 21 | MRPRESSO Outlier-corrected | 1.31 (1.13, 1.50) | 9.80E-04 |
|  | Depression | 21 | IVW | 1.02 (0.97, 1.08) | 4.32E-01 |
|  | Depression | 21 | Weighted median | 0.99 (0.93, 1.06) | 8.31E-01 |
|  | Depression | 21 | Weighted mode | 0.99 (0.91, 1.06) | 7.01E-01 |
|  | Depression | 21 | MR-Egger | 0.98 (0.85, 1.14) | 8.26E-01 |
|  | Depression | 21 | MRPRESSO Outlier-corrected | 1.00 (0.95, 1.05) | 9.60E-01 |
|  | Schizophrenia | 21 | IVW | 1.29 (1.11, 1.50) | 8.89E-04 |
|  | Schizophrenia | 21 | Weighted median | 1.26 (1.11, 1.43) | 2.92E-04 |
|  | Schizophrenia | 21 | Weighted mode | 1.24 (1.08, 1.42) | 1.76E-03 |
|  | Schizophrenia | 21 | MR-Egger | 0.92 (0.64, 1.31) | 6.42E-01 |
|  | Schizophrenia | 21 | MRPRESSO Outlier-corrected | 1.19 (1.08, 1.31) | 1.78E-03 |

# Table S6: Sensitivity analysis for bipolar disorder GWAS without UK Biobank participants

| Drugs | No of SNPs | Methods | OR, 95% CI | P value |
| --- | --- | --- | --- | --- |
| ACEIs | 1 | Wald ratio | 1.43 (1.02, 1.99) | 3.73E-02 |
| BBs | 6 | IVW | 1.07 (0.93, 1.23) | 3.57E-01 |
| CCBs | 24 | IVW | 1.06 (1.03, 1.09) | 1.76E-05 |

# Table S7: Sensitivity analysis using DBP-associated SNPs to proxy antihypertensive drugs in the European population

| Drug | Outcomes | No. of SNPs | Methods | OR, 95% CI | P value |
| --- | --- | --- | --- | --- | --- |
| ACEIs | Bipolar disorder | 1 | Wald ratio | 1.76 (1.03, 3.00) | 3.73E-02 |
|  | Depression | 1 | Wald ratio | 0.71 (0.56, 0.91) | 7.17E-03 |
|  | Schizophrenia | 1 | Wald ratio | 3.26 (1.99, 5.34) | 2.75E-06 |
| BBs | Bipolar disorder | 8 | IVW | 1.22 (0.91, 1.63) | 1.92E-01 |
|  | Bipolar disorder | 8 | Weighted median | 1.42 (0.97, 2.07) | 7.11E-02 |
|  | Bipolar disorder | 8 | Weighted mode | 1.44 (0.81, 2.58) | 2.15E-01 |
|  | Depression | 8 | IVW | 1.05 (0.92, 1.21) | 4.62E-01 |
|  | Depression | 8 | Weighted median | 1.05 (0.88, 1.25) | 5.83E-01 |
|  | Depression | 8 | Weighted mode | 1.09 (0.82, 1.46) | 5.63E-01 |
|  | Schizophrenia | 8 | IVW | 0.89 (0.63, 1.26) | 5.17E-01 |
|  | Schizophrenia | 8 | Weighted median | 1.05 (0.72, 1.53) | 8.15E-01 |
|  | Schizophrenia | 8 | Weighted mode | 1.08 (0.59, 2.00) | 8.01E-01 |
|  | Bipolar disorder | 38 | IVW | 1.51 (1.23, 1.85) | 6.66E-05 |
| CCBs | Bipolar disorder | 38 | Weighted median | 1.47 (1.19, 1.81) | 3.29E-04 |
|  | Bipolar disorder | 38 | MR-Egger | 1.24 (0.70, 2.20) | 4.56E-01 |
|  | Bipolar disorder | 38 | Weighted mode | 1.37 (0.87, 2.15) | 1.76E-01 |
|  | Bipolar disorder | 38 | MRPRESSO  Outlier-corrected | 1.51 (1.24, 1.83) | 1.28E-04 |
|  | Depression | 38 | IVW | 1.05 (0.98, 1.13) | 1.81E-01 |
|  | Depression | 38 | Weighted median | 0.98 (0.90, 1.07) | 7.12E-01 |
|  | Depression | 38 | MR-Egger | 1.01 (0.82, 1.25) | 9.21E-01 |
|  | Depression | 38 | Weighted mode | 0.98 (0.85, 1.12) | 7.59E-01 |
|  | Depression | 38 | MRPRESSO  Outlier-corrected | 1.04 (0.97, 1.12) | 2.98E-01 |
|  | Schizophrenia | 38 | IVW | 1.46 (1.23, 1.75) | 2.03E-05 |
|  | Schizophrenia | 38 | Weighted median | 1.49 (1.24, 1.80) | 2.63E-05 |
|  | Schizophrenia | 38 | MR-Egger | 1.05 (0.65, 1.71) | 8.34E-01 |
|  | Schizophrenia | 38 | Weighted mode | 1.83 (1.26, 2.65) | 1.53E-03 |
|  | Schizophrenia | 38 | MRPRESSO  Outlier-corrected | 1.51 (1.30, 1.76) | 2.62E-06 |
